# Action-guilt, survivor-guilt, and depression in combat-related PTSD

**DOI:** 10.1371/journal.pone.0351689

**Published:** 2026-07-02

**Authors:** Mika Allyssa Esquillo, Riya Kumar, Elizabeth Ellen Morris, Christina Bass, Mary Turner, Gail Tillman, John Hart Jr, Michael A. Motes

**Affiliations:** 1 Callier Center for Communication Disorders, School of Behavioral and Brain Sciences, University of Texas at Dallas, Richardson, Texas, United States of America; 2 Department of Psychology, University of Texas Southwestern Medical Center, Dallas, Texas, United States of America; 3 Department of Neurology, University of Texas Southwestern Medical Center, Dallas, Texas, United States of America; Fondazione Policlinico Universitario Agostino Gemelli IRCCS, Universita’ Cattolica del Sacro Cuore, ITALY

## Abstract

Identifying the role of guilt in post-traumatic stress disorder (PTSD) has important implications for understanding the development and treatment of PTSD. The present study is a secondary analysis of data collected on veterans with combat-related PTSD (N = 61) who enrolled in a clinical trial. Hierarchical regression analyses were used to explore associations between action- and survivor-guilt on the Clinician Administered PTSD Scale (CAPS), PTSD symptom severity on the CAPS, and depression on the Quick Inventory of Depressive Symptomatology (QIDS). Action- and survivor-guilt independently predicted PTSD total symptom severity, but in examining PTSD symptom cluster severity, only action-guilt independently predicted avoidance and numbing severity in PTSD. However, depression symptom severity on QIDS also mediated the association between action-guilt and PTSD total symptom severity and avoidance and numbing severity. Thus, action- and survivor-guilt appear to have independent contributions to PTSD symptom severity, and action-guilt, more specifically, appears to affect avoidance and numbing severity in PTSD through depression. The findings are discussed in terms of the potential role of guilt in PTSD and in relation to previous research on guilt and depression in PTSD and PTSD treatment.

## Introduction

Controversy exists regarding the specific role of guilt in PTSD [1]. Yet, the role of guilt in PTSD potentially has important implications for understanding the etiology of PTSD and for developing targeted treatments. Indeed, guilt has been postulated to affect recovery in PTSD [2], and therapy-based reductions in trauma-related guilt have been shown to mediate reductions in PTSD symptom severity and depression [3–8]. Thus, the present study sought to further explicate the role of guilt in PTSD in a sample of veterans with combat-related PTSD. This was a secondary analysis conducted using data collected at baseline on combat veterans with PTSD enrolled in a PTSD treatment trial [9].

Meta-analyses have shown moderate effect-sizes for the association between guilt and PTSD symptom severity (*r* = 0.38 [8]; *r* = 0.42 [10]). However, the strength of associations between guilt and PTSD symptom severity has also been shown to vary across general classes of index traumas and the types of guilt assessed [10,11]. Stronger effect-sizes have been observed for PTSD index traumas related to war/combat (*r* = 0.44 and *r* = 0.47), sexual assault (*r* = 0.40), and interpersonal violence (*r* = 0.32 to 0.42) than for traumas related childhood sexual abuse (*r* = 0.21) and motor vehicle accidents (*r* = 0.18) [10,11]. Additionally, stronger effect-sizes have been observed for assessments of trauma-specific guilt, *r* = 0.43, and general state-guilt (i.e., guilt not linked to specific contexts), *r* = 0.49, compared to guilt proneness (i.e., guilt experienced across contexts), *r* = 0.13 [11], and among Vietnam veterans, association between trauma-specific guilt and PTSD symptom severity has been found to be strong, 0.70 ≤ *r* ≤ 0.81, including, across intrusion [*r* = 0.80] and avoidance [*r* = 0.66] symptom clusters in PTSD [12], though also varying in strength across components of guilt (i.e., global guilt, distress, and guilt cognitions), 0.26 ≤ *r* ≤ 0.73 [13,14].

Early DSM diagnostic criteria for PTSD included guilt, specifically, Criterion D(3) – *Guilt about surviving while others have not or about behavior required for survival* [15, 16]. However, later DSM definitions included guilt as part of a set of associated features in PTSD rather than as part of the diagnostic criteria [16], and the Clinician Administered PTSD Scale (CAPS) for DSM-IV [17,18] also included guilt among a set of “associated features” and included items distinguishing between survivor-guilt and action-guilt. Survivor-guilt involves self-directed negative thoughts and emotions regarding surviving when others did not or regarding not being seriously injured when others were, and action guilt involves self-directed negative thoughts and emotions regarding perceived acts of commission or omission that led to the death or serious injury of others. In addition to CAPS for DSM-IV [17,18], other guilt scales have included items assessing survivor- and action-guilt [12,19,20], and models of PTSD in combat veterans have proposed that these foci of guilt can lead to different PTSD clinical syndromes, manifest in differences in emotions, cognitions, self-concepts, and relationships [21,22]. Although survivor- and action-guilt have different experiential foci, association between survivor- and action-guilt has been observed in combat veterans with PTSD [19], and the association suggests the possibility of a single, common “guilt” construct, rather than separable independent constructs, in PTSD. However, the association also has been interpreted as possibly occurring due to the complexities of index traumas and general combat exposure among combat veterans (i.e., often experiencing traumatic scenarios potentially triggering both survivor- and action-guilt) [19]. Yet, although correlated, potential unique contributions of survivor- and action-guilt in predicting PTSD symptom severity have not been explored.

Although both theoretical and clinical models have implicated guilt in the formation of PTSD, debate exists regarding the nature of the association between guilt and PTSD [1]. Though associated with PTSD, guilt also has been observed to be a significant feature in depression [23], and depression has been observed to commonly co-occur with PTSD [24,25], particularly, numbing in PTSD [26, 27, 28, but see 29]. Thus, guilt might be associated with PTSD through its role in depression, and the depression-mediated relationship might be specifically related to numbing in PTSD. However, alternative models have been put forward, including models arguing that 1) guilt is a causal mechanism mediating association between a trauma inducing event and PTSD, 2) PTSD is a causal mechanism mediating association between trauma and guilt, 3) guilt is an epiphenomenal correlate of PTSD with trauma as a causal mechanism for both, and 4) guilt is associated to PTSD through a mediating mechanism, like, depression [for review, see 1].

The present study aimed to further examine the potential role of guilt in PTSD by examining associations between trauma-related guilt, depression, and PTSD symptom severity. For this study, a secondary analysis was conducted using data collected at baseline on combat veterans with PTSD enrolled in a treatment trial examining the pairing of repeated transcranial magnetic stimulation (rTMS) with Cognitive Processing Therapy (CPT) to treat PTSD [9, see also 30]. The original study examined change in PTSD symptom severity on CAPS for DSM-IV [17,18], as the primary outcome, and change in depression on the Quick Inventory of Depression Symptomology (QIDS) [31], as a secondary outcome measure. Trauma-related guilt was measured via Associated Feature items on CAPS [17,18]. Although the Associated Feature data were collected in the original study, which included the items assessing survivor- and action-guilt, they were not used in the analyses in the published clinical trial results [9]. Thus, for the present study, PTSD symptom severity was assessed using the CAPS for DSM-IV [17,18]; guilt was assessed using the Associated Features items addressing survivor- and action-guilt; and depression was assessed using QIDS-SR16 [31].

## Materials and methods

### Participants

The sample consisted of US military veterans (N = 61; N = 4 females; see **Table 1** for additional demographic characteristics). All participants were diagnosed with PTSD based on a combat-related index trauma and were deployed in combat regions from 2001 forward, except for n = 2 who were not enrolled in the original treatment trial [9]. Participants were recruited from the community, primarily, the Dallas-Fort Worth Metroplex, for an rTMS + CPT treatment trial [9], conducted between 2011 and 2016, in an unbiased fashion with respect to race, ethnicity, and gender (ClinicalTrials.gov ID NCT01391832). Written informed consent was obtained from each participant prior to data collection, and all data were protected by a Certificate of Confidentiality. The original study [9] was approved by and complied with the University of Texas Southwestern Medical Center Institutional Review Board (IRB Number: STU-092010–035) and the University of Texas at Dallas (UTD) IRB (IRB Number: 10–57), and continuing analyses of the data were approved by the UTD IRB (IRB Number: IRB-24–245).

**Table 1:** Demographic Characteristics.

Male ^a^ (Proportion)		0.934
Age ^a^ (*M*, *SE*)		33.16 (0.900)
Education ^b^ (*M*, *SE*)		14.78 (0.274)
WASI IQ ^g^ (*M*, *SE*)		109.29 (1.702)
Race ^b^ (Proportion)		
	White	0.738
	African American	0.197
	Other	0.033
Hispanic ^a^ (Proportion)		0.180
Married ^b^ (Proportion)		0.492
Work ^c^ (Proportion)		
	Fulltime	0.361
	Unemployed	0.393
Student ^b^ (Proportion)		0.541
Branch ^e^ (Proportion)		
	Army	0.492
	Navy	0.0492
	Marines	0.311
	Air Force	0.0656
	Other	0.0164
Rank ^d^ (Proportion Enlisted)		0.836
VA Disability ^d^ (Proportion)		0.590
Years Active Duty ^c^ (*M*, *SE*) Combat Exposure FCES ^f^ (*M*, *SE*)		7.28 (0.635) 50.182 (3.490)
		
CAPS Total ^a^ (*M*, *SE*)		75.213 (2.600)
CAPS B ^a^ (*M*, *SE*)		18.738 (0.924)
CAPS C ^a^ (*M*, *SE*)		29.623 (1.193)
Avoidance (*M*, *SE*)		9.590 (0.560)
Numbing (*M*, *SE*)		17.066 (0.722)
CAPS D ^a^ (*M*, *SE*)		26.853 (0.850)
CAPS Survivor-Guilt ^a^ (*M*, *SE*)		2.803 (0.375)
CAPS Action-Guilt ^a^ (*M*, *SE*)		3.918 (0.362)
QIDS ^d^ (*M*, *SE*)		11.810 (0.599)

M, mean; SE, standard error; WASI IQ, Wechsler Abbreviated Scale of Intelligence Quotient; VA, Veterans’ Administration; FCES, Full Combat Exposure Scale; CAPS, Clinician Administered PTSD Scale; QIDS, Quick Inventory of Depressive Symptomology.

^a^n = 61

^b^N = 60

^c^N = 59

^d^N = 58

^e^N = 57

^f^N = 55

^g^N = 41

All participants were diagnosed with PTSD based on combat-related index traumas using CAPS for DSM-IV, based on the F1/I2 scoring rule [18], administered by trained clinicians. At the beginning of the original clinical trial, the Associated Features items from CAPS were not administered. Thus, although N = 103 veterans were enrolled in the original clinical trial, Associated Features data were only available on N = 49 of those participants. The additional data included in the present secondary analysis were obtained on participants (N = 12) who received a diagnosis of PTSD based on a combat-related index trauma at baseline but could not commit to completion of the full study following the baseline assessments (n = 10), had contraindications for consideration for participation in the full study (n = 1), or did not meet era criteria (n = 2). Permission to use the data collected in analyses during the consenting process was attained regardless of full completion of the study.

Participants were excluded from the original trial for: a history of psychiatric comorbidities, including, eating disorders, psychotic symptoms, current (less than 3 months) substance dependence or abuse; a history of a significant neurological/medical issues such as seizure, traumatic brain injury (moderate or severe TBI), brain tumors, stroke, blood vessel abnormalities in the brain, dementia, Parkinson’s disease, Huntington’s chorea, multiple sclerosis, cardiac pacemaker, implanted medication pumps of any sort, history of significant heart disease (e.g., myocardial infarction, tachyarrhythmia, congestive heart failure, valvular disease), or any metal objects in or near the head (most dental work was allowed) which could not be safely removed for TMS treatments. Veterans were also excluded if unable or unwilling to stop taking a prescription medication (e.g., stimulants) or illegal substances (e.g., cocaine) that significantly lower seizure thresholds. Female veterans who were pregnant or breastfeeding were also excluded because of a lack of data regarding rTMS/MRI safety during pregnancy or breastfeeding. Non-English speakers were also excluded because some of the screening forms, questionnaires, and tests were only available in English. Finally, participants could not start any new psychological treatment for PTSD while being in the study.

### Assessments

CAPS, based on the DSM-IV [15], was administered to participants by trained clinicians to assess PTSD symptom severity [17,18]. CAPS consisted of 17 items, addressing symptom frequency and intensity over the prior month, which were summed to yield a total severity score [18]. Three symptom cluster scales were addressed within the 17 items: CAPS B Re-Experiencing, CAPS C Avoidance/Numbing, and CAPS D Hyperarousal. CAPS has shown evidence of good reliability (e.g., internal consistency Total Severity Cronbach’s α = 0.95, and test-retest *r* = 0.89 to 1.00), convergent validity with other assessments of PTSD and trauma exposure (e.g., with Mississippi PTSD scale, *r* = 0.77, SCID PTSD scale, *r* = 0.89, Combat Exposure Scale *r* = 0.53), and clinical utility [32]. In addition to the 17 items addressing the three symptom clusters, items addressing action- and survivor-guilt from the “associated features” items in CAPS were administered. Action-guilt items addressed the percentage of time and the intensity of guilt experienced related to acts of commission or omission related to the index trauma (i.e., *Have you felt guilty about anything that you did or didn’t do during [EVENT]? How much of the time have you felt that way in the past month?; How strong were the feelings of guilt? How much distress or discomfort did they cause?)*, and survivor-guilt items addressed the percentage of time and the intensity of any guilt experienced related to surviving the index trauma when others did not (i.e., *Have you felt guilty about surviving [EVENT] when others did not? How much of the time have you felt that way in the past month? How strong were these feelings of guilt? How much distress or discomfort did they cause?*). Finally, depression was assessed on QIDS-SR16 [31]. QIDS-SR16 used a 0–3 scale on each of the 16 items to measure the severity of depressive symptoms. A higher score represented greater depressive symptom severity. QIDS also has shown good internal consistency (Cronbach’s α = 0.86) and convergent validity with other assessments of depression (e.g., with 30-item Inventory of Depressive Symptomatology *r* = 0.96 and Hamilton Rating Scale for Depression *r* = 0.86) [31].

### Analysis Plan

The data were analyzed using hierarchical regression modeling. Hierarchical regression allowed for assessment of the unique contributions of CAPS Survivor-Guilt, CAPS Action-Guilt, and QIDS in predicting PTSD symptom severity on CAPS. First, the unique contributions of CAPS Survivor- and Action-Guilt in predicting CAPS total symptom severity were evaluated, including evaluating the potential mediating influence of QIDS. Next, the unique contributions of CAPS Survivor- and Action-Guilt in predicting CAPS re-experiencing, avoidance/ numbing, and hyperarousal symptom cluster scores were evaluated, also including evaluating the potential mediating influence of QIDS. For the regression analyses, no evidence of residuals deviating from normality was observed (i.e., no evidence of outliers having undue influence, heteroscedasticity, or curvilinearity).

## Results

### Predicting PTSD symptom severity

Evaluations of associations between the variables of interest were conducted to obtain necessary evidence for mediation testing [33], that is assessments of unique associations between 1) CAPS Total and both Action- and Survivor Guilt, 2) CAPS Total and QIDS, and 3) QIDS and Action- and Survivor Guilt. First, both Action-Guilt, β = 0.357, *t*(60)=3.026, *p* = 0.004, and Survivor-Guilt, β = 0.242, *t*(60)=2.050, *p* = 0.045, were significant predictors of CAPS Total (Table 2, Model 1). Although Action-Guilt was the stronger predictor, Survivor-Guilt remained a significant predictor after entering Action-Guilt into the model first (i.e., statistically controlling for Action-Guilt). Thus, Action- and Survivor-Guilt both uniquely contributed to predicting CAPS Total. Next, QIDS was a significant predictor of CAPS Total, β = 0.541, *t*(57)=4.767, *p* < 0.001. Then, although Survivor-Guilt also was a significant predictor of QIDS *t*(57)= 2.062, *p =* 0.044, after entering Action-Guilt into the model, Survivor-Guilt was no longer a significant predictor, β = 0.198, *t*(57)= 1.619, *p = ns*. Finally, Action-Guilt also was a significant predictor of QIDS, β = 0.406, *t*(57)= 3.321, *p =* 0.002, and Action-Guilt remained a significant predictor even when Survivor-Guilt was entered into the model first, β = 0.369, *t*(57)= 3.017, *p* = 0.04 (**Table 2**, Model 2).

**Table 2:** Hierarchical Regression Analysis of CAPS Total and QIDS as functions of Survivor- and Action-Guilt and of QIDS as a mediator of the relationship between CAPS Total and Survivor- and Action-Guilt.

Regression Model			Hierarchical Model Step Statistics					Final Model Predictor Statistics	
Predicted Variable	Hierarchical Step	Predictor Added	*R* ^*2*^	*ΔR* ^*2*^	*df* Residual	Model *F*	*ΔR*^*2*^ *F*	β	*t*
1. CAPS Total	1	Action-Guilt	0.157		59	11.002 ^b^		0.357	3.026 ^b^
	Final	Survivor-Guilt	0.214	0.057	58	7.900 ^b^	4.201^a^	0.242	2.050 ^a^
2. QIDS	1	Survivor-Guilt	0.071		56	4.250 ^a^		0.198	1.619
	Final	Action-Guilt	0.203	0.132	55	6.983 ^b^	9.102 ^b^	0.369	3.017 ^b^
3. CAPS Total	1	QIDS	0.402		56	37.715 ^b^		0.541	4.767 ^b^
	2	Survivor-Guilt	0.435	0.033	55	21.171^b^	3.167	0.178	1.687
	Final	Action-Guilt	0.446	0.011	54	14.481^b^	1.057	0.114	1.028

CAPS, Clinician Administered PTSD Scale; QIDS, Quick Inventory of Depressive Symptomology; df, Degrees of freedom; Model *F, R², df Residual* are for each regression model after the indicated predictor was added; βs and *t*-values are for the final model after adding the predictor; regressor *df* = 1 for all *ΔR*^*2*^ *F*-tests, and regressor *df*s equal the number of model predictors for all model *F*-tests; full statistics for each hierarchical regression step are in **S1 Table in S1 File**.

^a^ p < 0.05

^b^ p < 0.01

Thus, although Survivor-Guilt predicted depression on QIDS, the association was not independent of the association with Action-Guilt; whereas, Action-Guilt uniquely contributed to predicting depression on QIDS. Thus, the combinations of associations suggested possible mediation of the relationship between action-guilt and PTSD symptom severity by depression [33].

Hierarchical modeling was then used to examine the potential mediating role of QIDS on the association between Action-Guilt and CAPS Total. When QIDS was entered into the hierarchical regression model first followed by Survivor-Guilt and then Action-Guilt, Action-Guilt was no longer a significant predictor of CAPS Total, β = 0.178, *t*(57)= 1.687, *p = ns* and β = 0.114, *t*(57)= 1.028, *p = ns*, respectively (**Table 2**, Model 3). For all regression models, see **S1 Table in S1 File**. Thus, the attenuation is consistent with depression mediating the association between action-guilt and PTSD symptom severity.

### Predicting PTSD symptom cluster severity

Next, hierarchical regression analyses were conducted to test for unique associations between Action- and Survivor-Guilt and severity on the PTSD symptom clusters of Re-Experiencing (CAPS B), Avoidance/Numbing (CAPS C), and Hyperarousal (CAPS D) and to test for potential mediation by QIDS. In the first step in the hierarchical modeling, the criterion symptom cluster of interest was regressed on the other two symptom clusters (i.e., controlling for the other two symptom clusters, first, and thus testing for the unique effects of guilt on the criterion cluster). Next, Survivor-Guilt was entered, and Action-Guilt was entered in the last step. In these sets of analyses examining guilt in predicting unique portions of variance in each symptom cluster, only Avoidance/Numbing was found to be significantly predicted by guilt and only by Action-Guilt, β = 0.233, *t*(60)=2.439, 𝚫*R*^2^ = 0.048, 𝚫*F*(1,56)=5.946, *p* = 0.018 (Table 3 Model 2). For all regression models, see S2-S4 Tables in S1 File. Thus, Action-Guilt uniquely contributed to predicting Avoidance/Numbing beyond the other CAPS cluster scales and Survivor-Guilt.

**Table 3:** Hierarchical Regression Analysis of CAPS Symptom Clusters as a function of Survivor- and Action-Guilt.

Regression Model			Hierarchical Model Step Statistics					Final Model Predictor Statistics	
Predicted Variable	Hierarchical Step	Predictor(s) Added	*R* ^*2*^	*ΔR* ^*2*^	*df* Residual	Model *F*	*ΔR*^*2*^ *F*	β	*t*
1. CAPS B	1	CAPS C	0.533		58	33.162 ^b^		0.318	2.484 ^a^
		CAPS D						0.496	4.166 ^b^
	2	Survivor-Guilt	0.535	0.002	57	21.875 ^b^	0.208	−0.044	−0.454
	Final	Action-Guilt	0.535	0.000	56	16.124 ^b^	0.009	0.010	0.095
2. CAPS C	1	CAPS B	0.474		58	26.082 ^b^		0.312	2.484 ^a^
		CAPS D						0.321	2.512 ^a^
	2	Survivor-Guilt	0.496	0.022	57	18.678 ^b^	2.510	0.133	1.419
	Final	Action-Guilt	0.544	0.048	56	16.711 ^b^	5.946 ^a^	0.233	2.439 ^a^
3. CAPS D	1	CAPS B	0.547		58	35.039 ^b^		0.477	4.166 ^b^
		CAPS C						0.315	2.512 ^a^
	2	Survivor-Guilt	0.552	0.005	57	23.435 ^b^	0.650	0.075	0.797
	Final	Action-Guilt	0.552	0.000	56	17.269 ^b^	0.002	0.004	0.041

CAPS, Clinician Administered PTSD Scale; QIDS, Quick Inventory of Depressive Symptomology; df, Degrees of freedom; model *F, R², df Residual* are for each regression model after the indicated predictor was added; βs, SEs, and *t*-values are for the final model after adding Action Guilt; Regressor *df* = 1 for all *ΔR*^*2*^ *F*-tests, and regressor *df*s equal the number of model predictors for all model *F*-tests; full statistics for each hierarchical regression step are in the **S2** to **S4 Tables in S1 File**.

^a^p < 0.05

^b^p < 0.001

Next, QIDS was added to the modeling to test for mediation by depression. When Action-Guilt was added after QIDS was entered into the model, Action-Guilt was no longer a significant predictor of Avoidance/Numbing, β = 0.17, *t*(57)=1.630, 𝚫*R*^2^ = 0.024, 𝚫*F*(1,52)=2.656, *p* = 0.109 (**Table 4** Model 1). For all regression models, see **S5 Table in S1 File**. Thus, as with the analysis of CAPS Total symptom severity, the association between Avoidance/Numbing and Action-Guilt was mediated by QIDS.

**Table 4:** Hierarchical Regression Analysis CAPS C, Avoidance, and Numbing as Functions of Survivor and Action Guilt.

Regression Model			Hierarchical Model Step Statistics					Final Model Predictor Statistics	
Predicted Variable	Hierarchical Step	Predictor(s) Added	*R* ^*2*^	*ΔR* ^*2*^	*df* Residual	Model *F*	*ΔR*^*2*^ *F*	β	*t*
1. CAPS C	1	CAPS B	0.432		55	20.908 ^b^		0.271	2.049 ^a^
		CAPS D						0.219	1.610
	2	QIDS	0.486	0.054	54	17.013 ^b^	5.671 ^a^	0.202	1.636
	3	Survivor-Guilt	0.510	0.024	53	13.772 ^b^	2.569	0.150	1.494
	Final	Action-Guilt	0.533	0.024	52	11.893 ^b^	2.656	0.170	1.630
2. Avoidance	1	CAPS B	0.382		58	17.929 ^b^		0.311	2.179 ^a^
		CAPS D						0.288	1.981
	2	Survivor-Guilt	0.394	0.012	57	12.378 ^b^	1.170	0.104	0.969
	Final	Action-Guilt	0.410	0.016	56	9.728 ^b^	1.471	0.132	1.213
3. Numbing	1	CAPS B	0.318		58	13.541 ^b^		0.321	2.232 ^a^
		CAPS C						0.164	1.119
	2	Survivor-Guilt	0.355	0.036	57	10.438 ^b^	3.204	0.176	1.639
	Final	Action-Guilt	0.404	0.050	56	9.497 ^b^	4.661 ^a^	0.235	2.159 ^a^
		CAPS B						0.261	1.746
4. Numbing	1	CAPS D	0.348		54	9.588 ^b^		0.047	0.308
		QIDS						0.237	1.707
	2	Survivor-Guilt	0.383	0.036	53	8.234 ^b^	3.069	0.186	1.650
	Final	Action-Guilt	0.408	0.025	52	7.170 ^b^	2.183	0.173	1.477
		CAPS B						0.239	1.638
5. Numbing	1	CAPS D	0.386		57	11.941 ^b^		0.088	0.596
		Avoidance						0.263	2.015
	2	Survivor-Guilt	0.410	0.024	56	9.729 ^b^	2.285	0.149	1.410
	Final	Action-Guilt	0.445	0.035	55	8.825 ^b^	3.483	0.201	1.866

CAPS, Clinician Administered PTSD Scale; QIDS, Quick Inventory of Depressive Symptomology; df, Degrees of freedom; Model *F, R², df Residual* are for each regression model after the indicated predictor was added; βs, SEs, and *t*-values are for the final model after adding Action Guilt; Regressor *df* = 1 for all *ΔR*^*2*^ *F*-tests, and regressor *df*s equal the number of model predictors for all model *F*-tests; Full statistics for each hierarchical regression step are in **S5** to **S9 Tables in S1 File**.

^a^p < 0.05

^b^p < 0.001

Given research providing evidence for separation of avoidance and numbing symptoms and differences in associations with depression [15,16,23], CAPS C Avoidance/Numbing was divided into Avoidance severity and Numbing severity, and separate analyses were conducted with each a criterion variable (**Table 4**, Models 2 & 3), and for all models, see **S6 and S7 Tables in S1 File**. After controlling for CAPS B and CAPS D, only Numbing was significantly predicted by Action-Guilt, β = 0.235, *t*(60)=2.159, 𝚫*R*^2^ = 0.050, 𝚫*F*(1,56)=4.661, *p* = 0.035. However, after entering QIDS into the model, Action-Guilt was no longer a significant predictor of Numbing, β = 0.173, *t*(57)=1.477, 𝚫*R*^2^ = 0.025, 𝚫*F*(1,52)=2.183, *p* = 0.146 (**Table 4**, Model 4). Additionally, after entering Avoidance into the model, Action-Guilt was also no longer a significant predictor of Numbing, β = 0.201, *t*(60)=1.866, 𝚫*R*^2^ = 0.035, 𝚫*F*(1,55)=3.483, *p* = 0.146 (**Table 4**, Model 5). For all regression models, see S8 and S9 Tables in S1 File, respectively. Thus, although evidence for depression-mediated association between Action-Guilt and Numbing was found, the final result suggests that Action-Guilt cannot be determined to affect Numbing independently of Avoidance.

## Discussion

The present findings align with previous research showing associations between guilt and PTSD [1], guilt and depression [13], and depression and PTSD [14]. However, the present findings provide a more nuanced examination of the associations between PTSD severity and survivor- and action-guilt related to the PTSD inducing event and of the mediating role of depression. The findings support separable influences of survivor- and action-guilt on PTSD severity and suggest action-guilt could be specifically affecting avoidance and numbing symptoms in PTSD. However, the findings also suggest that the depression mediates the influences of action-guilt on PTSD and on avoidance and numbing.

Action- and survivor-guilt independently predicted PTSD symptom severity, and action-guilt independently predicted avoidance and numbing symptom severity. Thus, although correlated, possibly due to complex traumatic events and to combat exposure in total [e.g., 12,24], the separable contributions of action- and survivor-guilt in predicting PTSD symptom severity provides evidence for unique contributions of guilt stemming from negative self-directed thoughts and emotions regarding surviving or not being seriously injured in a traumatic event (i.e., survivor-guilt) versus self-directed negative thoughts and emotions regarding acts of commission or omission (i.e., action-guilt) on PTSD severity. However, findings from the present study also support mediation of the association between action-guilt and PTSD severity by depression, suggesting that action-guilt affects PTSD through co-morbid depression. Although survivor-guilt, independently of action-guilt, also predicted PTSD severity, survivor-guilt did not predict depression independently of action-guilt. Thus, the association between survivor-guilt and depression might be due to a more general form of guilt or might be through association with action-guilt due to complex trauma exposure [19].

Action-guilt also independently predicted avoidance and numbing symptoms in PTSD in the present study. Research has suggested that avoidance and numbing are defining features of PTSD [27,34], and research also has provided evidence for avoidant coping as a mediator of trauma-related guilt and PTSD symptom severity, at least, in interpersonal violence [35]. However, in the present study, depression also was observed to mediate the association between action-guilt and PTSD symptom severity and the association between action-guilt and avoidance/numbing symptom severity. Thus, the findings from the present study suggest that the more specific influence of action-guilt on avoidance/numbing is through co-morbid depression.

The present findings support and extend previous research on associations between combat-related guilt, depression, and PTSD [36,37]. In separate models, guilt cognitions have been shown to have direct effects and indirect effects via guilt-related distress on PTSD, and guilt cognitions also have been shown to have direct effects and indirect effects via guilt-related distress on depression [37]. In a combined model, combat-related guilt has been shown to mediate associations between abusive violence in war and both PTSD and Major Depressive Disorder (MDD), where PTSD and MDD were modeled as separate but correlated constructs [36]. Then, present findings suggest that combat-related action- and survivor-guilt affect co-morbid depression in PTSD. Furthermore, in a study examining the association between atrocity exposure in war and suicidal ideation, guilt was observed to have direct mediating effects, mediating effects through PTSD, mediating effects through depression, and mediating effects through depression associated with PTSD [38]. Thus, guilt may be driving component of co-morbid depression in PTSD, with action-guilt having unique influence on depression related to PTSD and with particular components of guilt, like, action-guilt, having stronger, depression-mediated influences particular PTSD symptom clusters, like, avoidance and numbing.

A mechanism by which guilt might be associated with PTSD is through introspective mental processes mediated by the Default Mode Network (DMN). Meta-analyses have shown disruptions of DMN resting-state connectivity in PTSD [39,40], and treatment response in PTSD also has been associated with DMN changes [41]. Meta-analysis also has shown increased DMN activity during experimental conditions targeting rumination or engagement in self-referential thought [42]. Additionally, in depression, although meta-analyses have revealed mixed DMN resting-state hyper-connectivity [43] and hypoconnectivity [44,45], depressed patients have shown disruption in their ability to use non-self-referential reappraisal of negative emotional stimuli to modulate activity within DMN [46]. However, while DMN dysfunction seems to be a candidate mechanism, direct research is needed to examine DMN functioning as a mediator of the associations between action- and survivor-guilt and PTSD and between action-guilt mediated by depression in avoidance and numbing in PTSD identified in the current study.

Although the present findings support depression as a mediator of the association between action-guilt and avoidance and numbing in PTSD, debate still exists regarding the structure of core symptoms in PTSD [e.g., 13,14,47–51]. Factor analytic studies have raised questions about the number and structure of PTSD symptom clusters [e.g., 47–51], and in particular, evidence has suggested separation of numbing and avoidance in PTSD [49–51], Furthermore, research on dissociation between avoidance and numbing symptoms in PTSD suggests that numbing might result from hyperarousal [28,52] and that active avoidance might be in response to re-experiencing symptoms [52]. Although numbing might, in part, occur due to resource depletion or as a mechanism in response to hyperarousal [28,52], the present findings suggest that action-guilt, mediated by depression, is also a unique contributor to avoidance and numbing in PTSD. That is, the association between action-guilt and avoidance and numbing was observed even after statistically controlling for hyperarousal and re-experiencing. However, action-guilt was not found to influence avoidance independent of numbing and vice versa, and thus, the findings suggest that action-guilt might affect shared rather than independent components of avoidance and numbing.

With interventions targeting trauma-related guilt having shown efficacy for reducing trauma-related guilt, depression, and PTSD symptom severity [2,3,5], the present findings suggest that targeting maladaptive cognitions separately underlying survivor- and action-guilt might have independent beneficial effects on PTSD symptom reduction. Although survivor- and action-guilt items on CAPS specifically address guilt frequency and intensity, cognitions and beliefs related to perceived culpability are components of guilt in PTSD (e.g., hindsight bias, preventability, violation of personal standards, and lack of justification [2,13,14]), and in CAPS-5 [53] guilt has been added to the diagnostic criteria (Item D4) on a symptom cluster assessing negative alterations in cognitions and mood that also includes items addressing cognitive distortions (Items D2 & D3). With both survivor- and action-guilt, the maladaptive cognitions and beliefs can be persecutorial and overaccommodative (e.g., with action-guilt, thinking that obsessive focus on wrongdoing will prevent future incidents or that living with never-ending guilt is simply the price for atonement or a way to honor those harmed; with survivor-guilt, obsessive focus on inequity in having survived when others did not and feeling less deserving of happiness [or living] due to the inequity and as a form of atonement) [54], but the unique contributions of survivor- and action-guilt to predicting PTSD symptom severity, observed in the present study, suggests some unique, mediating maladaptive cognitions can arise and contribute to PTSD symptom severity and can then be separately targeted for PTSD symptom reduction. In CPT integrating trauma accounts (CPT-A), the therapist and client use the index trauma account to gain an understanding the nature of a patient’s guilt and maladaptive cognitions. Thus, although survivor- and action-guilt severity are explicitly addressed on CAPS Associated Features items, during CPT, clearer expressions of guilt associated with index trauma events and outcomes are developed through discussions and targeted questions about the index trauma, stuck-points, and homework (e.g., expressions in the impact statement and worksheets, like, the A-B-C Worksheets focusing on connecting [A] Activating Event, [B] Belief/Cognitions, and [C] Consequences/Feelings and the Challenging Beliefs Worksheet that explicitly asks for ratings of emotions regarding stuck points), all making clearer to the therapist and client the associations between events and patterns of thinking contributing to survivor- and action-guilt. From these sources, the therapist can then target cognitive distortions and help the patient engage in cognitive restructuring and facilitate the creation of a narrative that fosters transition from avoidance/numbing to acceptance—key elements in CPT [54,55]. Furthermore, although both action- and survivor-guilt were associated with avoidance/numbing in the present study, the observed unique association between action-guilt and avoidance/numbing and mediating effect of depression suggests that avoidance/numbing and comorbid depression might create greater therapeutic challenges with index-traumas triggering action-guilt, but the associations also suggest that successful therapeutic intervention changing maladaptive cognitions in action-guilt could more strongly affect avoidance/numbing which could then benefit other later-engaged therapeutic processes.

### Limitations

Various aspects of the present research need to be considered with respect to limitations to generalization of the findings from the study. This was a secondary analysis of data collected for the treatment trial [6], and thus, for example, hypotheses were not formulated, measures used were not selected, and statistical power estimates were not obtained for this secondary analysis prior to data collection. The unique study selection pressures also might limit the generalizability of the observed findings regarding guilt, depression, and PTSD. The data were from a sample of participants enrolled in a treatment trial for combat-related PTSD [7], that is, predominantly white male combat veterans from the OEF/OIF era of combat, with other specific demographic characteristics (e.g., English-speaking), who elected to enroll in the treatment trial. In meta-analyses, for example, the type of index trauma has been shown to influence the association between guilt and PTSD symptom severity [10,11], with index traumas related to combat and war having among the strongest associations between guilt and PTSD severity but comparable association strengths for sexual assault and interpersonal violence index traumas. Although comparable in associative strength, the potential mediating role of depression also has yet to be examined for other index traumas. Additionally, although the present study examined survivor- and action-guilt related to combat-related index traumas, other research has examined other components of guilt (i.e., distress, guilt cognitions, and global guilt [e.g., 37]; hindsight bias, wrongdoing, and lack of justification [e.g., 56]) in PTSD. Furthermore, pre- and peri-trauma factors (e.g., personality [57], culture/social support [11], prior trauma [35,58], and coping style/strategies [35]) have been shown to be associated with guilt in PTSD. Demographic factors for the present sample were examined for potential mediation of the observed effects (see **S10 Table in S1 File**), but only combat exposure (i.e., FCES Total [59]) was found to be significantly correlated with PTSD symptom severity (i.e., CAPS Total) [see also 60,61] and survivor-guilt, but not avoidance/numbing (i.e., CAPS C) nor action-guilt. Yet, formal mediation testing via hierarchical regression, revealed that survivor-guilt remained a significant predictor of PTSD symptom severity even after controlling for combat exposure (see **S11 Table in S1 File**; for CAPS C analysis, see **S12 Table in S1 File**). However, future research including broader demographic assessments, a wider range of index traumas, and more detailed assessments of guilt will allow for more comprehensive evaluations of guilt profiles in PTSD. Finally, given the correlational designs used in the present and previous studies, caution should be used in making strong causal inferences regarding guilt in PTSD. Indeed, treatment studies have shown reductions in guilt predict future reductions in PTSD severity [3,4,6,62,63], and meta-analysis has provided evidence of guilt predicting PTSD development longitudinally [10], with both findings providing additional evidence for directional inference in guilt contributing to PTSD severity. Yet, guilt also has been proposed and modeled as a mediator between PTSD and suicidal ideation [64,65], that is, PTSD contributes to suicidal ideation via guilt.

## Conclusions

The present study provided evidence of survivor- and action-guilt as independent predictors of combat-related PTSD symptom severity among a group of combat veterans, and the study provided evidence of depression mediating the association between combat-related PTSD and action-guilt, and more specifically, depression mediating the association between action-guilt and avoidance and numbing symptoms in PTSD. These findings add to the understanding of trauma-related guilt in combat-related PTSD, in particular, showing differentiation of the contributions of survivor- and action-guilt to PTSD severity and thus contributing to research on guilt profiles in PTSD [21,22,66]. Guilt assessments can be used to aid in identifying potential leverage points in therapeutic interventions targeting cognitive reappraisals [e.g., 22,54,55,66,67]. With evidence preliminarily suggesting that interventions aimed at trauma-related guilt have efficacy in reducing trauma-related guilt, depression, and PTSD symptom severity [2,3,5], the present findings suggest that targeting maladaptive cognitions separately underlying survivor- and action-guilt might have independent beneficial effects on PTSD symptom reduction, and changing maladaptive cognitions related to action-guilt, in particular, might lead to reduced avoidance and numbing and thus benefit other later-engaged therapeutic processes.

## Supporting information

S1 File Supplementary Materials: Regression Tables. (DOCX)
