# Supplementary material for: Action-guilt, survivor-guilt, and depression in combat-related PTSD
Source: PLoS One. 2026 Jul 2;21(7):e0351689. doi: 10.1371/journal.pone.0351689 (PMC13327125; doi:10.1371/journal.pone.0351689)
Supplement: S1 File — (DOCX) [file pone.0351689.s001.docx]

**Supplementary Materials**

| S1 Table. Hierarchical regression of CAPS Total on QIDS, Survivor Guilt, and Action Guilt | | | | | | | | |  | |
| --- | --- | --- | --- | --- | --- | --- | --- | --- | --- | --- |
| Hierarchical Step | Model | *R^2^* | *ΔR^2^* | *df* Residual | β | *t* | Model *F* | *ΔR^2^* *F* | |  |
|  | Constant |  |  |  |  | 7.430 ^b^ |  |  | |  |
| 1 | QIDS | 0.402 |  | 56 | 0.634 | 6.141 ^b^ | 37.715 ^b^ |  | |  |
|  | Constant |  |  |  |  | 7.343 ^b^ |  |  | |  |
|  | QIDS |  |  |  | 0.585 | 5.562 ^b^ |  |  | |  |
| 2 | Survivor Guilt | 0.435 | 0.033 | 55 | 0.187 | 1.780 | 21.171 ^b^ | 3.167 | |  |
|  | Constant |  |  |  |  | 7.201 ^b^ |  |  | |  |
|  | QIDS |  |  |  | 0.541 | 4.767 ^b^ |  |  | |  |
|  | Survivor Guilt |  |  |  | 0.178 | 1.687 |  |  | |  |
| Final | Action Guilt | 0.446 | 0.011 | 54 | 0.114 | 1.028 | 14.481 ^b^ | 1.057 | |  |

CAPS, Clinician Administered PTSD Scale; QIDS=Quick Inventory of Depression Symptomology; Regressor *df* = 1 for

all *ΔR^2^* *F*-tests, and regressor *df*s equal the number of model predictors for all model *F*-tests.

^a^ *p* < 0.05

^b^ *p* < 0.01

| S2 Table. Hierarchical regression of CAPS B on CAPS C, CAPS D, Survivor Guilt, and Action Guilt | | | | | | | | |
| --- | --- | --- | --- | --- | --- | --- | --- | --- |
| Hierarchical  Step | Model | *R^2^* | *ΔR^2^* | *df* Residual | β | *t* | Model *F* | *ΔR^2^* *F* |
|  | Constant |  |  |  |  | -1.000 |  |  |
|  | CAPS C |  |  |  | 0.311 | 2.669 ^a^ |  |  |
| 1 | CAPS D | 0.533 |  | 58 | 0.491 | 4.209 ^b^ | 33.162 ^b^ |  |
|  | Constant |  |  |  |  | -1.022 |  |  |
|  | CAPS C |  |  |  | 0.322 | 2.689 ^b^ |  |  |
|  | CAPS D |  |  |  | 0.496 | 4.204 ^b^ |  |  |
| 2 | Survivor Guilt | 0.535 | 0.002 | 57 | -0.044 | -0.456 | 21.875 ^b^ | 0.208 |
|  | Constant |  |  |  |  | -1.012 |  |  |
|  | CAPS C |  |  |  | 0.318 | 2.484 ^a^ |  |  |
|  | CAPS D |  |  |  | 0.496 | 4.166 ^b^ |  |  |
|  | Survivor Guilt |  |  |  | -0.044 | -0.454 |  |  |
| Final | Action Guilt | 0.535 | 0.000 | 56 | 0.010 | 0.095 | 16.124 ^b^ | 0.009 |

CAPS, Clinician Administered PTSD Scale; Regressor *df* = 1 for all *ΔR^2^* *F*-tests, and regressor *df*s equal the number of model predictors for all model *F*-tests.

^a^ *p* < 0.05

^b^ *p* < 0.01

| S3 Table. Hierarchical regression of CAPS C on CAPS B, CAPS D, Survivor Guilt, and Action Guilt | | | | | | | | | |
| --- | --- | --- | --- | --- | --- | --- | --- | --- | --- |
| Hierarchical  Step | Model | *R^2^* | *ΔR^2^* | *df* Residual | β | *t* | Model *F* | *ΔR^2^* *F* |  |
|  | Constant |  |  |  |  | 1.664 |  |  |  |
|  | CAPS B |  |  |  | 0.351 | 2.669 ^a^ |  |  |  |
| 1 | CAPS D | 0.474 |  | 58 | 0.397 | 3.015 ^b^ | 26.082 ^b^ |  |  |
|  | Constant |  |  |  |  | 1.731 |  |  |  |
|  | CAPS B |  |  |  | 0.349 | 2.689 ^b^ |  |  |  |
|  | CAPS D |  |  |  | 0.357 | 2.697 ^b^ |  |  |  |
| 2 | Survivor Guilt | 0.496 | 0.022 | 57 | 0.155 | 1.584 | 18.678 ^b^ | 2.510 |  |
|  | Constant |  |  |  |  | 1.643 |  |  |  |
|  | CAPS B |  |  |  | 0.312 | 2.484 ^a^ |  |  |  |
|  | CAPS D |  |  |  | 0.321 | 2.512 ^a^ |  |  |  |
|  | Survivor Guilt |  |  |  | 0.133 | 1.419 |  |  |  |
| Final | Action Guilt | 0.544 | 0.048 | 56 | 0.233 | 2.439 ^a^ | 16.711 ^b^ | 5.946 ^a^ |  |

CAPS, Clinician Administered PTSD Scale; Regressor *df* = 1 for all *ΔR^2^* *F*-tests, and regressor *df*s equal the number of

model predictors for all model *F*-tests.

^a^ *p* < 0.05

^b^ *p* < 0.01

| S4 Table. Hierarchical regression of CAPS D on CAPS B, CAPS C, Survivor Guilt, and Action Guilt | | | | | | | | |
| --- | --- | --- | --- | --- | --- | --- | --- | --- |
| Hierarchical Step | Model | *R^2^* | *ΔR^2^* | *df* Residual | β | *T* | Model *F* | *ΔR^2^* *F* |
|  | Constant |  |  |  |  | 5.700 ^b^ |  |  |
|  | CAPS B |  |  |  | 0.477 | 4.209 ^b^ |  |  |
| 1 | CAPS C | 0.547 |  | 58 | 0.341 | 3.015 ^b^ | 35.039 ^b^ |  |
|  | Constant |  |  |  |  | 5.693 ^b^ |  |  |
|  | CAPS B |  |  |  | 0.477 | 4.204 ^b^ |  |  |
|  | CAPS C |  |  |  | 0.317 | 2.697 ^b^ |  |  |
| 2 | Survivor Guilt | 0.552 | 0.005 | 57 | 0.075 | 0.806 | 23.435 ^b^ | 0.650 |
|  | Constant |  |  |  |  | 5.643 ^b^ |  |  |
|  | CAPS B |  |  |  | 0.477 | 4.166 ^b^ |  |  |
|  | CAPS C |  |  |  | 0.315 | 2.512 ^a^ |  |  |
|  | Survivor Guilt |  |  |  | 0.075 | 0.797 |  |  |
| Final | Action Guilt | 0.552 | 0.000 | 56 | 0.004 | 0.041 | 17.269 ^b^ | 0.002 |

CAPS, Clinician Administered PTSD Scale; Regressor *df* = 1 for all *ΔR^2^* *F*-tests, and regressor *df*s equal the number of

model predictors for all model *F*-tests.

^a^ *p* < 0.05

^b^ *p* < 0.01

|  |  |  |  | |  |  |  |  | |  | |  |  | |  |
| --- | --- | --- | --- | --- | --- | --- | --- | --- | --- | --- | --- | --- | --- | --- | --- |
| S5 Table. Hierarchical regression of CAPS C on CAPS B, CAPS D, QIDS, Survivor Guilt, and Action Guilt | | | | | | | | | | | | | |  |  |
| Hierarchical Step | Model | *R^2^* | | *ΔR^2^* | *df* Residual | β | *t* | | Model *F* | | *ΔR^2^* *F* | |  | | |
|  | Constant |  | |  |  |  | 2.000 | |  | |  | |  | | |
|  | CAPS B |  | |  |  | 0.364 | 2.646 ^a^ | |  | |  | |  | | |
| 1 | CAPS D | 0.432 | |  | 55 | 0.355 | 2.583 ^a^ | | 20.908 ^b^ | |  | |  | | |
|  | Constant |  | |  |  |  | 1.782 | |  | |  | |  | | |
|  | CAPS B |  | |  |  | 0.282 | 2.069 ^a^ | |  | |  | |  | | |
|  | CAPS D |  | |  |  | 0.253 | 1.828 | |  | |  | |  | | |
| 2 | QIDS | 0.486 | | 0.054 | 54 | 0.287 | 2.381 ^a^ | | 17.013 ^b^ | | 5.671 ^a^ | |  | | |
|  | Constant |  | |  |  |  | 1.918 | |  | |  | |  | | |
|  | CAPS B |  | |  |  | 0.283 | 2.104 ^a^ | |  | |  | |  | | |
|  | CAPS D |  | |  |  | 0.218 | 1.576 | |  | |  | |  | | |
|  | QIDS |  | |  |  | 0.262 | 2.193 ^a^ | |  | |  | |  | | |
| 3 | Survivor Guilt | 0.510 | | 0.024 | 53 | 0.163 | 1.603 | | 13.772 ^b^ | | 2.569 | |  | | |
|  | Constant |  | |  |  |  | 1.858 | |  | |  | |  | | |
|  | CAPS B |  | |  |  | 0.271 | 2.049 ^a^ | |  | |  | |  | | |
|  | CAPS D |  | |  |  | 0.219 | 1.610 | |  | |  | |  | | |
|  | QIDS |  | |  |  | 0.202 | 1.636 | |  | |  | |  | | |
|  | Survivor Guilt |  | |  |  | 0.150 | 1.494 | |  | |  | |  | | |
| Final | Action Guilt | 0.533 | | 0.024 | 52 | 0.170 | 1.630 | | 11.893 ^b^ | | 2.656 | |  | | |

CAPS, Clinician Administered PTSD Scale; QIDS=Quick Inventory of Depression Symptomology; Regressor *df* = 1 for all

*ΔR^2^* *F*-tests, and regressor *df*s equal the number of model predictors for all model *F*-tests.

^a^ *p* < 0.05

^b^ *p* < 0.01

| S6 Table. Hierarchical regression of CAPS Avoidance on CAPS B, CAPS C, Survivor Guilt, and Action Guilt | | | | | | | | | |
| --- | --- | --- | --- | --- | --- | --- | --- | --- | --- |
| Hierarchical Step | Model | *R^2^* | *ΔR^2^* | *df* Residual | β | *t* | Model *F* | *ΔR^2^* *F* |  |
|  | Constant |  |  |  |  | -0.102 |  |  |  |
|  | CAPS B |  |  |  | 0.334 | 2.343 ^a^ |  |  |  |
| 1 | CAPS D | 0.382 |  | 58 | 0.338 | 2.372 ^a^ | 17.929 ^b^ |  |  |
|  | Constant |  |  |  |  | -0.070 |  |  |  |
|  | CAPS B |  |  |  | 0.333 | 2.336 ^a^ |  |  |  |
|  | CAPS D |  |  |  | 0.308 | 2.127 ^a^ |  |  |  |
| 2 | Survivor Guilt | 0.394 | 0.012 | 57 | 0.116 | 1.082 | 12.378 ^b^ | 1.170 |  |
|  | Constant |  |  |  |  | -0.149 |  |  |  |
|  | CAPS B |  |  |  | 0.311 | 2.179 ^a^ |  |  |  |
|  | CAPS D |  |  |  | 0.288 | 1.981 |  |  |  |
|  | Survivor Guilt |  |  |  | 0.104 | 0.969 |  |  |  |
| Final | Action Guilt | 0.410 | 0.016 | 56 | 0.132 | 1.213 | 9.728 ^b^ | 1.471 |  |

CAPS, Clinician Administered PTSD Scale; Regressor *df* = 1 for all *ΔR^2^* *F*-tests, and regressor *df*s equal the number

of model predictors for all model *F*-tests.

^a^ *p* < 0.05

^b^ *p* < 0.01

|  |  |  |  | | |  |  | |  | |  |  | |  | |  | |
| --- | --- | --- | --- | --- | --- | --- | --- | --- | --- | --- | --- | --- | --- | --- | --- | --- | --- |
| S7 Table. Hierarchical regression of CAPS Numbing on CAPS B, CAPS C, Survivor Guilt, and Action Guilt | | | | | | | | | | | | | | | | |  |
| Hierarchical Step | Model | *R^2^* | | *ΔR^2^* | *df* Residual | | β | *t* | | Model *F* | | | *ΔR^2^* *F* | |  |  |  |
|  | Constant |  | |  |  | |  | 2.370 ^a^ | |  | | |  | |  |  |  |
|  | CAPS B |  | |  |  | | 0.361 | 2.410 ^a^ | |  | | |  | |  |  |  |
| 1 | CAPS D | 0.318 | |  | 58 | | 0.251 | 1.676 | | 13.341 ^b^ | | |  | |  |  |  |
|  | Constant |  | |  |  | |  | 2.467 ^a^ | |  | | |  | |  |  |  |
|  | CAPS B |  | |  |  | | 0.358 | 2.438 ^a^ | |  | | |  | |  |  |  |
|  | CAPS D |  | |  |  | | 0.2 | 1.336 | |  | | |  | |  |  |  |
| 2 | Survivor Guilt | 0.321 | | 0.036 | 57 | | 0.198 | 1.79 | | 10.438 ^b^ | | | 3.204 | |  |  |  |
|  | Constant |  | |  |  | |  | 2.400 ^a^ | |  | | |  | |  |  |  |
|  | CAPS B |  | |  |  | | 0.321 | 2.232 ^a^ | |  | | |  | |  |  |  |
|  | CAPS D |  | |  |  | | 0.164 | 1.119 | |  | | |  | |  |  |  |
|  | Survivor Guilt |  | |  |  | | 0.176 | 1.639 | |  | | |  | |  |  |  |
| Final | Action Guilt | 0.404 | | 0.050 | 56 | | 0.235 | 2.159 ^a^ | | 9.497 ^b^ | | | 4.661 ^a^ | |  |  |  |

CAPS, Clinician Administered PTSD Scale; Regressor *df* = 1 for all *ΔR^2^* *F*-tests, and Regressor dfs equal the

number of model predictors for all model *F*-tests.

^a^ *p* < 0.05

^b^ *p* < 0.01

| S8 Table. Hierarchical regression of CAPS Numbing on CAPS B, CAPS D, QIDS, Survivor Guilt, and Action Guilt | | | | | | | | | |
| --- | --- | --- | --- | --- | --- | --- | --- | --- | --- |
| Hierarchical Step | Model | *R^2^* | *ΔR^2^* | *df* Residual | β | *t* | Model *F* | *ΔR^2^* *F* |  |
|  | Constant |  |  |  |  | 2.472 ^a^ |  |  |  |
|  | CAPS B |  |  |  | 0.271 | 1.766 |  |  |  |
|  | CAPS C |  |  |  | 0.089 | 0.571 |  |  |  |
| 1 | QIDS | 0.348 |  | 54 | 0.329 | 2.425 ^a^ | 9.588 ^b^ |  |  |
|  | Constant |  |  |  |  | 2.638 ^a^ |  |  |  |
|  | CAPS B |  |  |  | 0.272 | 1.806 |  |  |  |
|  | CAPS C |  |  |  | 0.046 | 0.296 |  |  |  |
|  | QIDS |  |  |  | 0.299 | 2.229 ^a^ |  |  |  |
| 2 | Survivor Guilt | 0.383 | 0.036 | 53 | 0.2 | 1.752 | 8.234 ^b^ | 3.069 |  |
|  | Constant |  |  |  |  | 2.585 ^a^ |  |  |  |
|  | CAPS B |  |  |  | 0.261 | 1.746 |  |  |  |
|  | CAPS C |  |  |  | 0.047 | 0.308 |  |  |  |
|  | QIDS |  |  |  | 0.237 | 1.707 |  |  |  |
|  | Survivor Guilt |  |  |  | 0.186 | 1.650 |  |  |  |
| Final | Action Guilt | 0.408 | 0.025 | 52 | 0.173 | 1.477 | 7.170 ^b^ | 2.183 |  |

CAPS, Clinician Administered PTSD Scale; QIDS=Quick Inventory of Depression Symptomology; Regressor *df* = 1 for all *ΔR^2^* *F*-tests, and regressor *df*s equal the number of model predictors for all model *F*-tests.

^a^ *p* < 0.05

^b^ *p* < 0.01

|  |  |  |  |  |  |  |  | |  | |  | |  | |
| --- | --- | --- | --- | --- | --- | --- | --- | --- | --- | --- | --- | --- | --- | --- |
| S9 Table. Hierarchical regression of CAPS Numbing on CAPS Avoidance, CAPS B, CAPS D, Survivor Guilt, and Action Guilt | | | | | | | | | | | | | |  |
| Hierarchical Step | Model | *R^2^* | *ΔR^2^* | *df* Residual | β | *t* | | Model *F* | | *ΔR^2^* *F* | |  |  |  |
|  | Constant |  |  |  |  | 2.509 ^a^ | |  | |  | |  |  |  |
|  | CAPS B |  |  |  | 0.250 | 1.669 | |  | |  | |  |  |  |
|  | CAPS C |  |  |  | 0.139 | 0.926 | |  | |  | |  |  |  |
| 1 | CAPS Avoidance | 0.386 |  | 57 | 0.331 | 2.505 ^a^ | | 11.941 ^b^ | |  | |  |  |  |
|  | Constant |  |  |  |  | 2.579 ^a^ | |  | |  | |  |  |  |
|  | CAPS B |  |  |  | 0.258 | 1.737 | |  | |  | |  |  |  |
|  | CAPS C |  |  |  | 0.107 | 0.711 | |  | |  | |  |  |  |
|  | CAPS Avoidance |  |  |  | 0.303 | 2.293 ^a^ | |  | |  | |  |  |  |
| 2 | Survivor Guilt | 0.410 | 0.024 | 56 | 0.163 | 1.512 | | 9.729 ^b^ | | 2.285 | |  |  |  |
|  | Constant |  |  |  |  | 2.504 ^a^ | |  | |  | |  |  |  |
|  | CAPS B |  |  |  | 0.239 | 1.638 | |  | |  | |  |  |  |
|  | CAPS C |  |  |  | 0.088 | 0.596 | |  | |  | |  |  |  |
|  | CAPS Avoidance |  |  |  | 0.263 | 2.015 ^a^ | |  | |  | |  |  |  |
|  | Survivor Guilt |  |  |  | 0.149 | 1.410 | |  | |  | |  |  |  |
| Final | Action Guilt | 0.445 | 0.035 | 55 | 0.201 | 1.866 | | 8.825 ^b^ | | 3.483 | |  |  |  |

CAPS, Clinician Administered PTSD Scale; Regressor *df* = 1 for all *ΔR^2^* *F*-tests, and regressor *df*s equal the number of model predictors for all model *F*-tests.

^a^ *p* < 0.05

^b^ *p* < 0.01

S10 Table. Correlation matrix for CAPS, QIDS, and demographics variables.

|  | CAPS Total | CAPS B | CAPS C | CAPS D | Survivor-Guilt | Action-Guilt | QIDS |
| --- | --- | --- | --- | --- | --- | --- | --- |
| CAPS B | 0.868 ^bc^ |  |  |  |  |  |  |
| CAPS C | 0.890 ^bc^ | 0.625 ^bc^ |  |  |  |  |  |
| CAPS D | 0.866 ^bc^ | 0.690 ^bc^ | 0.639 ^bc^ |  |  |  |  |
| CAPS Survivor-Guilt | 0.300 ^ac^ | 0.191 ^c^ | 0.316 ^ac^ | 0.267 ^ac^ |  |  |  |
| CAPS Action-Guilt | 0.396 ^ac^ | 0.285 ^ac^ | 0.436 ^bc^ | 0.290 ^ac^ | 0.163 ^c^ |  |  |
| QIDS | 0.634 ^bf^ | 0.523 ^bf^ | 0.572 ^bf^ | 0.546 ^bf^ | 0.266 ^af^ | 0.406 ^af^ |  |
| Age | 0.119 ^c^ | 0.064 ^c^ | 0.129 ^c^ | 0.114 ^c^ | 0.078 ^c^ | 0.080 ^c^ | 0.135 ^f^ |
| Education (Years) | -0.168 ^d^ | -0.140 ^d^ | -0.189 ^d^ | -0.096 ^d^ | -0.103 ^d^ | -0.044 ^d^ | -0.065 ^f^ |
| WASI IQ | -0.320 ^aj^ | -0.158 ^j^ | -0.310 ^aj^ | -0.350 ^aj^ | -0.100 ^j^ | -0.209 ^j^ | -0.180 ^j^ |
| Race (1=White) | -0.040 ^e^ | 0.061 ^e^ | -0.125 ^e^ | -0.014 ^e^ | -0.070 ^e^ | -0.048 ^e^ | -0.116 ^g^ |
| Ethnicity (1=Hispanic) | -0.111 ^e^ | -0.112 ^e^ | -0.086 ^e^ | -0.097 ^e^ | -0.049 ^e^ | 0.033 ^e^ | -0.075 ^g^ |
| Marital Status (1=Married) | -0.019 ^c^ | 0.082 ^c^ | -0.077 ^c^ | -0.038 ^c^ | -0.204 ^c^ | 0.029 ^c^ | -0.109 ^f^ |
| Branch (1=Army) | 0.112 ^g^ | 0.121 ^g^ | 0.082 ^g^ | 0.098 ^g^ | 0.128 ^g^ | -0.150 ^g^ | 0.179 ^i^ |
| Rank (1=Officer) | -0.107 ^e^ | -0.106 ^e^ | -0.103 ^e^ | -0.071 ^e^ | 0.031 ^e^ | 0.060 ^e^ | 0.012 ^g^ |
| % VA Disability | -0.182 ^f^ | -0.270 ^af^ | -0.139 ^f^ | -0.069 ^f^ | 0.030 ^f^ | -0.061 ^f^ | -0.059 ^h^ |
| Years Active Duty | -0.040 ^e^ | 0.009 ^e^ | -0.085 ^e^ | -0.013 ^e^ | -0.015 ^e^ | 0.007 ^e^ | 0.005 ^g^ |
| Deployments (Years) | 0.225 ^e^ | 0.183 ^e^ | 0.202 ^e^ | 0.208 ^e^ | 0.132 ^e^ | 0.121 ^e^ | 0.017 ^g^ |
| FCES (Total) | 0.290 ^ai^ | 0.424 ^ai^ | 0.195 ^i^ | 0.140 ^i^ | 0.296 ^ai^ | 0.200 ^i^ | 0.056 ^i^ |

Codes for categorical demographic variables: Race 1=White, 0=all others; Ethnicity 1=Hispanic, 0=Non-hispanic; Marital status 1=Married, 0=all others; Branch 1=Army, 0=all others; Rank 1=Officer, 0=Enlisted. CAPS=Clinician Administered PTSD Scale; QIDS=Quick Inventory of Depressive Symptomalogy; WAIS IQ=Wechsler’s Adult Intelligence Scale Intelligence Quotient; VA=Veteran’s Administration; FCES=Full Combat Exposure Scale

^a^ *p* < 0.05

^b^ *p* < 0.01

^c^ *n* = 61

^d^ *n* = 60

^e^ *n* = 59

^f^ *n* = 58

^g^ *n* =57

^h^ *n* =56

^i^ *n* = 55

^j^ *n* = 41

Correlations of participant demographics with CAPS measures and QIDS were examined (including dummy-coding for demographic variables as noted, **S10 Table**). The demographic variables examined were not associated with QIDS nor Action-Guilt. However, Full Combat Exposure Scale (FCES) was significantly correlated with CAPS Total, CAPS B, and Survivor-Guilt. IQ was also significantly associated with CAPS Total, CAPS C, and CAPS D, but not Survivor- or Action-Guilt. Thus, given the associations between FCES, CAPS Total, and Survivor-Guilt, potential mediation of the association between CAPS Total and Survivor-Guilt by FCES was tested (see **S11 Table**). Additionally, based on the findings reported in the manuscript on association between CAPS C and Survivor- and Action-Guilt, potential mediation by FCES also was examined (see **S12 Table**).

S11 Table. Hierarchical regression of CAPS Total on FCES, Survivor Guilt, and Action Guilt

| Hierarchical Step | Model | *R^2^* | *ΔR^2^* | *df* Residual | Model *F* | *ΔR^2^* *F* | β | *t* |  |
| --- | --- | --- | --- | --- | --- | --- | --- | --- | --- |
|  | Constant |  |  |  |  |  | 63.480 | 11.180^b^ |  |
| 1 | FCES | 0.084 |  | 53 | 4.853^a^ |  | 0.290 | 2.203 ^a^ |  |
|  | Constant |  |  |  |  |  | 61.071 | 10.816 ^b^ |  |
|  | FCES |  |  |  |  |  | 0.209 | 1.565 |  |
| 2 | Survivor-Guilt | 0.151 | 0.067 | 52 | 4.614^a^ | 4.092^a^ | 0.271 | 2.023 ^a^ |  |
|  | Constant |  |  |  |  |  | 55.294 | 9.574 ^b^ |  |
|  | FCES |  |  |  |  |  | 0.157 | 1.226 |  |
|  | Survivor-Guilt |  |  |  |  |  | 0.224 | 1.748 |  |
| Final | Action-Guilt | 0.253 | 0.102 | 51 | 5.763^b^ | 6.996^a^ | 0.330 | 2.645^a^ |  |

CAPS, Clinician Administered PTSD Scale; FCES=Full Combat Exposure Scale; Regressor *df* = 1 for all *ΔR^2^* *F*-tests, and

regressor *df*s equals the number of model predictors for all model *F*-tests.

^a^ *p* < 0.05

^b^ *p* < 0.01

Given the observed associations between CAPS Total, FCES, and Survivor Guilt, hierarchical modeling was used to examine the potential mediating role of FCES on the association between Survivor- and Action-Guilt with CAPS Total. When FCES was entered into the model first (**S11 Table**, Step 1) followed by Survivor-Guilt (**S11 Table**, Step 2), Survivor-Guilt was a significant predictor of CAPS Total, with *ΔR^2^*=0.067, β=0.271, *t*(52)= 2.023, *p*<0.05. Then when Action-Guilt was added to the model (**S11 Table**, Final Step), Action-Guilt also was a significant predictor of CAPS Total, with *ΔR^2^*=0.102, β=0.330, *t*(51)=2.645, *p*<0.05, but as in the modeling with just Survivor-Guilt and Action-Guilt, Survivor-Guilt was no longer a significant predictor of CAPS Total, with β=0.224, *t*(51)=1.748, *p*<0.05=*ns*. Thus, both Survivor- and Action-Guilt predicted PTSD symptom severity after controlling for combat exposure.

S12 Table. Hierarchical regression of CAPS C on CAPS B, CAPS D, FCES, Survivor Guilt, and Action Guilt

| Hierarchical Step | Model | *R^2^* | *ΔR^2^* | *df* Residual | Model *F* | *ΔR^2^* *F* | β | *t* |
| --- | --- | --- | --- | --- | --- | --- | --- | --- |
|  | Constant |  |  |  |  |  | 4.892 | 1.226 |
|  | CAPS B |  |  |  |  |  | 0.353 | 2.655^a^ |
| 1 | CAPS D | 0.476 |  | 52 | 23.66^b^ | 23.66^b^ | 0.405 | 3.047^b^ |
|  | Constant |  |  |  |  |  | 5.081 | 1.180 |
|  | CAPS B |  |  |  |  |  | 0.361 | 2.411^a^ |
|  | CAPS D |  |  |  |  |  | 0.402 | 2.931^b^ |
| 2 | FCES | 0.477 | 0.000 | 51 | 15.48^b^ | 0.015 | -0.014 | -0.124 |
|  | Constant |  |  |  |  |  | 6.291 | 1.522 |
|  | CAPS B |  |  |  |  |  | 0.398 | 2.771^b^ |
|  | CAPS D |  |  |  |  |  | 0.323 | 2.399^a^ |
|  | FCES |  |  |  |  |  | -0.095 | -0.839 |
| 3 | Survivor Guilt | 0.534 | 0.057 | 50 | 14.322^b^ | 6.153^a^ | 0.259 | 2.481^a^ |
|  | Constant |  |  |  |  |  | 6.411 | 1.608 |
|  | CAPS B |  |  |  |  |  | 0.376 | 2.707^b^ |
|  | CAPS D |  |  |  |  |  | 0.279 | 2.129^a^ |
|  | FCES |  |  |  |  |  | -0.118 | -1.069 |
|  | Survivor Guilt |  |  |  |  |  | 0.240 | 2.373^a^ |
| Final | Action Guilt | 0.575 | 0.041 | 49 | 13.255^b^ | 4.722^a^ | 0.217 | 2.173^a^ |

CAPS, Clinician Administered PTSD Scale; FCES=Full Combat Exposure Scale; Regressor *df* = 1 for all *ΔR^2^* *F*-tests, and

regressor *df*s equal the number of model predictors for all model *F*-tests.

^a^ *p* < 0.05

^b^ *p* < 0.01

Given the observed associations between CAPS C, FCES, and Survivor Guilt, hierarchical modeling was used to examine the potential mediating role of FCES on the association between Survivor- and Action-Guilt with CAPS C, while controlling for CAPS B and D. When FCES was entered into the model first (**S12 Table**, Step 2) followed by Survivor-Guilt (**S12 Table**, Step 3), Survivor-Guilt was a significant predictor of CAPS Total, with *ΔR^2^*=0.057, β=0.259, *t*(50)= 2.481, *p*<0.05. Then, when Action-Guilt was added to the model (**S12 Table**, Final Step), Action-Guilt also was a significant predictor of CAPS C, with *ΔR^2^*=0.041, β=0.217,*t*(49)=2.173, *p*<0.05. Thus, both Survivor- and Action-Guilt predicted avoidance and numbing severity after controlling for combat exposure.
