# Supplementary material for: Action-guilt, survivor-guilt, and depression in combat-related PTSD
Source: PLoS One. 2026 Jul 2;21(7):e0351689. doi: 10.1371/journal.pone.0351689 (PMC13327125; doi:10.1371/journal.pone.0351689)
Supplement: Attachment — Submitted filename: Reply.docx [file pone.0351689.s003.docx]

**Reviewer #1**

*1. Introduction is well narrated but only focus on that guilt is a consequence of PTSD symptoms, rather than a cause of PTSD symptoms, but also some other literature supports the opposite. So better to touch both ideas in order to send message for future study.*

We have clarified in the revision the possibility raised here in the Introduction, on Lines 90-101, relying on the review by Pugh el al. [1], and in the Discussion, particularly, the Lines 315-329 and in the added Limitations sections, particularly, Lines 380-386.

*2. Better to show which guilty is in particular association with PTSD according to your finding?*

We have clarified the findings in the Abstract, Lines 34-40; in the Results with summary statements on Lines 206-208, 215-218, 226-227, 248-250, 254-255, and 270-283; and the Discussion on Lines 289-292, 300-306, 307-309, 313-314, 352-356, and in the Conclusions.

*3.* *Line 215-217 you explained the action guilty independently predicted PTSD symptom severity, so that better to explain whether the survivor guilt independently predicted PTSD symptom severity or not?*

As noted in Reply #2, we have clarified the associations in the revision. Particularly, on the issue raised here, when action- and survivor-guilt were entered into the regression model, without QIDS, (Table 2 Model 1), both were significant predictors of CAPS Total. Thus, as we have clarified on Lines 206-208:

“Although Action-Guilt was the stronger predictor, Survivor-Guilt remained a significant predictor after entering Action-Guilt into the model first (i.e., statistically controlling for Action-Guilt). Thus, Action- and Survivor-Guilt both uniquely contributed to predicting CAPS Total.”

However, Survivor-Guilt was not a significant predictor of QIDS independent of Action-Guilt, as summarized on Lines 215-218:

“Thus, although Survivor-Guilt predicted depression on QIDS, the association was not independent of the association with Action-Guilt; whereas, Action-Guilt uniquely contributed to predicting depression on QIDS.”

Thus, when examining for mediation by QIDS, Action-Guilt was the predictor of interest. Similarly, in the PTSD Cluster analyses, Action-Guilt was the predictor of interest. We also have clarified this in the Discussion as noted in Reply #2 above.

*4. Finally, you’re not adequately address the limitations of the study, I mean I can say there is no limitation of the study, so that please address it?*

As described above in Reply #2, we have added a limitations section, Lines 357-386.

**Reviewer #2**

*1. I believe it would greatly enhance the manuscript if the authors included a thorough discussion of the study's limitations. Addressing potential weaknesses or constraints of the research would provide a more balanced perspective.*

As noted in Reviewer 1 Reply #4 above, we have added discussion of the limitations on Lines 357-386.

*2. Additionally, it would be beneficial to incorporate specific recommendations for future research based on the findings presented. This would not only guide subsequent studies but also demonstrate the practical applications of the research. Furthermore, discussing the theoretical implications of the study’s results would enrich the readers' understanding of its significance within the broader academic discourse.*

We have added discussion on possible future research in the Limitations section noted above, explicitly in Lines 377-380, and more implicitly Lines 304-306, 326-329, DMN discussion 330-341, and in the Conclusion regarding applications to therapy Lines 395-402.

**Reviewer #3**

*1. The title accurately reflects the study's focus on the mediating role of depression in the relationship between guilt (both action and survivor guilt) and PTSD symptoms. The literature review is comprehensive, citing relevant studies that establish the connections between guilt, depression, and PTSD. However, while the references are generally up-to-date, some could benefit from more recent studies to enhance the literature's breadth and depth.*

We have added many more recent references in the revision, notably, two meta-analyses examining guilt and PTSD: [10] Kip et al (2022) doi: 10.1017/S0033291722001866, and [11] Shi et al (2021) doi: 10.1016/j.janxdis.2021.102443.

*2. The methodology section is detailed and provides essential information about the participant selection, assessment tools, and statistical analyses employed. However, it could benefit from a more explicit description of the exclusion criteria and the rationale behind the sample size. Additionally, while the use of the Clinician Administered PTSD Scale (CAPS) and the Quick Inventory of Depressive Symptomatology (QIDS) is appropriate, a brief discussion of their psychometric properties would strengthen the justification for their use.*

We have included summaries of the psychometric properties of the measures in the Assessments sub-section, Lines 167-189.

We also have included more information on the inclusion/exclusion criteria and sample size for the original treatment trial, Lines 151-166. Because the present manuscript is a secondary analysis of data collected for that treatment trial, we did not obtain *a priori* minimum sample size estimate for this secondary analysis. We used all of the data that were available. However, the cited meta-analysis [11] reported a low-moderate to moderate (0.37<r<0.49) confidence interval for the association between combat-related guilt and PTSD symptom severity. Using the lower end, with r=0.37, alpha =0.05, and power=0.80, the minimum sample-size needed would be N=11. We have added discussion on the study not initially being powered for this secondary analysis to the Limitations section, Lines 360-362, in the revision.

*3. The study excluded participants with certain psychiatric comorbidities and those who were not proficient in English. This could limit the generalizability of the findings to a broader population of veterans, particularly those who may have additional mental health challenges or language barriers.*

As noted above, we have included a Limitations sections in the revision, and that Limitations section includes references to these issues.

*4. The study is based on a secondary analysis of data collected for a different purpose (a clinical trial). This raises concerns about the original data's suitability for the current research questions. The lack of control over the initial data collection process may introduce biases or confounding variables that were not accounted for.*

As noted above, we have included a Limitations section discussing the issues raised here.

*5. The scales used in the study are appropriate for measuring PTSD symptoms and depression. The CAPS is a well-established tool for assessing PTSD, and the QIDS is a reliable measure of depressive symptoms. However, the article could enhance its rigor by discussing the specific aspects of these scales that align with the study's focus on guilt and its effects.*

We have elaborated on issues relevant to the reviewer’s point in the Introduction and Discussion. Particularly, in the Introduction, Lines 52-69, we discuss findings on different guilt constructs in relation to PTSD. Then in Lines 70-89, we have provided more in-depth discussion on survivor- and action-guilt in PTSD. Then, in the Discussion, Lines 315-329, in particular, we discuss the present findings in relation to other research on guilt, depression, and PTSD. Finally, in the Limitations section of the Discussion, we also note the limitations of our work to action- and survivor-guilt and that a more comprehensive view of guilt in PTSD would require examining other components of guilt (e.g., distress, guilt cognitions, global guilt, hindsight bias, wrongdoing, lack of justification, etc.).

*6. The statistical analyses employed, including hierarchical regression, are appropriate for the study's aims. The authors effectively demonstrate the mediating role of depression in the relationship between guilt and PTSD symptoms. However, a more detailed explanation of the assumptions underlying the regression analyses and how they were tested would improve the transparency of the statistical approach.*

We have noted, on Lines 198-200, that there was no concerning evidence of regression residuals deviating from normality (i.e., showing outliers having undue influence, heteroscedasticity, and curvilinearity). The following figures show residual plots for PTSD Total and Avoidance/Numbing from the full model regression analyses and show PTSD Total and QIDS residuals plotted as functions of predicted values for the variables of interest.

*7. The values presented in the tables align with the interpretations provided in the text. The article could benefit from a more explicit connection between specific statistical values and their implications for the study's. Moreover, the study does not appear to control for other potential confounding variables that could influence the relationships being studied, such as trauma history, social support, or coping strategies. These factors could significantly impact the severity of PTSD symptoms and the experience of guilt and depression.*

As noted above in our reply to Reviewer 1 #2, we have added statements in the results to make more explicit the connections between the statistical values and their implications, in the Results with summary statements on Lines 206-208, 215-218, 226-227, 248-250, 254-255, and 270-283. Additionally, in the Limitations section, we have added discussion on the potential influences of trauma history, social support, and coping strategies.

*8. The authors discuss the results in relation to previous literature, highlighting the unique contributions of action and survivor guilt to PTSD symptom severity. They effectively contextualize their findings within the broader research landscape. However, the discussion could be strengthened by addressing potential alternative explanations for the observed relationships and considering the implications of their findings for clinical practice.*

We have provided elaboration on alternative explanations in the Discussion, Lines 315-329, by discussing previous research on the guilt, depression, and PTSD. We elaborate on studies treating PTSD and depression as separate constructs [37], separate but correlated constructs [36,38]. Additionally, in the Limitations section, we note issues with deriving causal inference from the current study and other data from correlational designs.

*9. The study acknowledges several limitations, including the small sample size and the cross-sectional design, which limits causal inferences. The authors recommend future research to explore longitudinal relationships and the effectiveness of guilt-targeting interventions. However, they could further address the potential impact of comorbid conditions beyond depression and suggest specific strategies for future studies to enhance generalizability.*

We have added discussion on these points to the added Limitations section.

*10. Although the study suggests that guilt-targeting treatments may be beneficial, it does not provide specific recommendations for clinical practice or future research directions. A more thorough discussion of how these findings can be translated into effective interventions would enhance the study's impact.*

As noted in other answers above, we have provided more on recommendations for future research in various parts of the Discussion section. With respect to treatments and potential research on treatments, we felt that it was necessary to be cautious with arguments regarding intervention specifics, because we are not presenting treatment data. However, we have added suggestions in the revision on Lines 395-402, particularly, elaborating on our previously short statement on “leverage points.”
