# Supplementary material for: Action-guilt, survivor-guilt, and depression in combat-related PTSD
Source: PLoS One. 2026 Jul 2;21(7):e0351689. doi: 10.1371/journal.pone.0351689 (PMC13327125; doi:10.1371/journal.pone.0351689)
Supplement: Attachment — Submitted filename: response.docx [file pone.0351689.s004.docx]

*We would like to thank the reviewers for their time and attention in considering our manuscript. We are pleased that Reviewer 3 was satisfied with our responses and the previous revision and that Reviewer 2 was largely satisfied but added the following two suggestions.*

*1. The authors should provide specific recommendations for each identified limitation.*

Regarding limitations, in the current revision, we have added the following statement on recommendations, “Thus, future research including broader demographic assessments, a wider range of index traumas, and more detailed assessments of guilt will allow for more comprehensive evaluations of guilt profiles in PTSD in future research.” Additionally, we have revised the paragraph to read, “Finally, given the correlational designs used in the present and previous studies, caution should be used in making strong causal inferences regarding guilt in PTSD. Guilt also has been proposed and modeled as a mediator between PTSD and suicidal ideation [58,59], for example. However, treatment studies also have shown reductions in guilt predict future reductions in PTSD severity [3,4,6,60,61], and meta-analysis has provided evidence of guilt predicting PTSD development longitudinally [10], with both findings providing additional evidence for directional inference in guilt contributing to PTSD severity.”

*2. It would be beneficial for the authors to include a distinct section outlining the practical and theoretical implications of the study, rather than incorporating them into the conclusion section.*

We added the following paragraph to the Discussion to further elaborate on the practical and theoretical implications (lines 353-381):

With interventions targeting trauma-related guilt having shown efficacy for reducing trauma-related guilt, depression, and PTSD symptom severity [2, 3, 5], the present findings suggest that targeting maladaptive cognitions separately underlying survivor- and action-guilt might have independent beneficial effects on PTSD symptom reduction. Although action- and survivor-guilt items on CAPS specifically address guilt frequency and intensity, cognitions and beliefs related to perceived culpability are components of guilt in PTSD (e.g., hindsight bias, preventability, violation of personal standards, and lack of justification^2^), and in CAPS-5 [53] guilt has been added to the diagnostic criteria (Item D4) on a symptom cluster assessing negative alterations in cognitions and mood that also includes items addressing cognitive distortions (Items D2 & D3). With both survivor- and action-guilt, the maladaptive cognitions and beliefs can be persecutorial and overaccommodative (e.g., with action-guilt, thinking that obsessive focus on wrongdoing will prevent future incidents or that living with never-ending guilt is simply the price for atonement or a way to honor those harmed; with survivor-guilt, obsessive focus on inequity in having survived when others did not and feeling less deserving of happiness [or living] due to the inequity and as a form of atonement) [54] but the unique contributions of survivor- and action-guilt to predicting PTSD symptom severity, observed in the present study, suggests some unique, mediating maladaptive cognitions can arise and contribute to PTSD symptom severity and can then be separately targeted for PTSD symptom reduction. In CPT integrating trauma accounts (CPT-A), the therapist and client use the index trauma account to gain an understanding the nature of a patient’s guilt and maladaptive cognitions, and the therapist can the target cognitive distortions and help the patient engage in cognitive restructuring and facilitate the creation of a narrative that fosters transition from avoidance/numbing to acceptance—key elements in CPT [54,55]. Furthermore, although both action- and survivor guilt were associated with avoidance/numbing in the present study, the observed unique association between action-guilt and avoidance/numbing and mediating effect of depression suggests that avoidance/numbing and comorbid depression might create greater therapeutic challenges with index-traumas triggering action-guilt, but the associations also suggest that successful therapeutic intervention changing maladaptive cognitions in action-guilt could more strongly affect avoidance/numbing which could then benefit other later-engaged therapeutic processes.
