# Supplementary material for: Action-guilt, survivor-guilt, and depression in combat-related PTSD
Source: PLoS One. 2026 Jul 2;21(7):e0351689. doi: 10.1371/journal.pone.0351689 (PMC13327125; doi:10.1371/journal.pone.0351689)
Supplement: Attachment — Submitted filename: reply_r3.docx [file pone.0351689.s005.docx]

We thank the editor and reviewers for their consideration of our manuscript for publication in PLOS One. We also appreciate the opportunity to address the latest comments, and we feel that the revisions made in response have strengthened the manuscript. Below are our responses to the comments.

1. *The introduction could be more concise.*

We have condensed the introduction, particularly, paragraph 2.

2. *…the results section could better highlight key findings upfront*

We have modified the results section to clarify the order of testing presented. For mediation testing, one must first show associations among the variables of interest [33]. The first paragraph in the results section reports on this testing, and we added the following line to clarify:

(Line 203) “Evaluations of associations between the variables of interest were conducted to obtain necessary evidence for mediation testing [33], that is assessments of unique associations between 1) CAPS Total and both Action- and Survivor Guilt, 2) CAPS Total and QIDS, and 3) QIDS and Action- and Survivor Guilt.”

We also added the following line as a summary to further clarify the importance of the results presented:

(Line 227) “Thus, the combinations of associations suggested possible mediation of the relationship between action-guilt and PTSD symptom severity by depression [33].”

3. *Tables are detailed but could be simplified for readability (e.g., merging redundant models or adding a summary table).*

We have revised the Tables to improve readability. In particular, we have reformatted the tables to *highlight the* steps in the regression hierarchical models. In the tables, we have separated analyses with solid lines and noted in the first column the criterion variable examined in the analysis. Then, we separated steps in each hierarchical model with dashed lines, numbered the results from the steps tested, and labeled the row showing the results from the “Final” model. Then, to highlight that the Beta-weights and t-values are from the “Final” model tested in the different analyses, we moved those statistics to the ends of the tables, shaded them in gray, and labeled the columns “Final Model Predictor Statistics” (see Table 2 example below).

We hope that these changes have also addressed the reviewer’s parenthetical comment about model redundancy. We are not reporting redundant models in Tables 2, 3, & 4. Arguably, there is redundancy in the regression model results reported in the Supplementary Materials because of a replication of patterns of statistics from prior steps in the hierarchical analyses, but the purpose of the Supplementary Tables is to report the complete sets of statistics from the hierarchical modeling steps, including, showing the changes to the regression weights with the addition of new variables to the model.

4. *The sample is predominantly male (93.4%) and from a specific combat era (OEF/OIF), which may limit generalizability. The authors should explicitly discuss this and caution against overgeneralizing to other populations (e.g., civilians, female veterans, or veterans from other conflicts).*

We stated this in the limitations section:

(Line 398) “**The unique study selection pressures also might limit the generalization of the observed findings regarding guilt, depression, and PTSD.** The data were from a sample of participants enrolled in a treatment trial for combat-related PTSD [7], that is, predominantly white male combat veterans from the OEF/OIF era of combat, with other specific demographic characteristics (e.g., English-speaking), who elected to enroll in the treatment trial.”

*5. The small sample size (N = 61) and secondary analysis design (with pre-existing data constraints) should be framed as limitations.*

We stated this in the limitations section:

(Line 395) “This was a secondary analysis of data collected for the treatment trial [6], and thus, for example, hypotheses were not formulated, measures used were not selected, and statistical power estimates were not obtained for this secondary analysis prior to data collection.”

6. *The study design is correlational, and while mediation is tested, causal claims should be tempered. The discussion appropriately notes this, but the abstract and introduction could more clearly state that causal inferences are speculative.*

As noted by the reviewer in this comment, in previous submissions, we explicitly stated this in the limitations section:

(Line 422) “Finally, given the correlational designs used in the present and previous studies, caution should be used in making strong causal inferences regarding guilt in PTSD.”

The only wording that we could find in our abstract suggesting “causal inference” is stated speculatively:

(Line 38) Thus, action- and survivor-guilt appear to have independent contributions to PTSD symptom severity, and action-guilt, more specifically, appears to affect avoidance and numbing severity in PTSD through depression.

The wording used in our introduction also is appropriately speculative:

(Line 85) Yet, although correlated, **potential** unique contributions of survivor- and action-guilt in **predicting** PTSD symptom severity have not been explored.

(Line 99) The present study aimed to further examine the **potential role** of guilt in PTSD by examining associations between trauma-related guilt, depression, and PTSD symptom severity.

We could not find any cases where we made “strong” causal claims, but if we have missed such cases, we are certainly willing to temper them as we have done with the statements referenced above and with our clear caveats regarding causal inferences that we have made in the limitations section (Lines 422-424).

*7. The introduction could better integrate the theoretical models of guilt in PTSD (e.g., Kubany’s cognitive model) to ground the hypotheses.*

It is not clear to us how this Kubany’s work would ground our hypotheses regarding survivor- and action-guilt, that is, beyond the referencing of that work that we have already put in the manuscript. We discuss the theoretical models of Pugh et al. [1] in the Introduction to ground the hypotheses (Lines 87-98), and this is in addition to our discussion of testing for potential unique associations of survivor- and action-guilt with PTSD severity (Lines 79-86). Additionally, we do reference Kubany’s work in the introduction [13,14] and discussion [65] sections, in addition to other related research on guilt profiles [21,22] and leverage points in therapy. However, based on our reading of Kubany’s work, the models do not lead to predictions regarding separable influences of survivor- and action-guilt, but maybe we are just not clear on the reviewer’s point here with respect to the relevance of Kubany’s work.

In our reading of Kubany’s work [65], “hindsight bias” is at the core of a form of “I should have known better” guilt; whereas, “Catch 22 dilemma” is at the core of a form of guilt arising from experiencing moral violations based actions in war referenced to “peacetime” moral values (i.e., war forces combatants to engage in behavior that would be considered “antithetically contrary to deeply ingrained family and societal values…[related to]…nonviolence and preservation of life”). However, as Kubany notes explicitly and in the anecdotes provided in [65], hindsight biases and Catch 22 dilemmas can arise from action/inaction events, survivor events, and their combinations. For example, in discussing hindsight bias, the three examples are on guilt arising from failures to act (i.e., action/inaction events) that led to deaths of compatriots (i.e., survivor events) (p 6), and under Catch 22, three of the anecdotes are on guilt arising from action events (i.e., killing combatants when not wanting to; killing children; using a deceased buddy as a shield) and the fourth in an action/inaction and survivor event (i.e., retreating under orders and surviving when a friend was killed) (p 8). In our reading, guilt coming from hindsight bias and Catch 22 dilemmas could be survivor- or action-guilt [65].

Kubany also proposed what we would argue is an elaboration on the above model with global guilt, distress, and guilt cognition components, particularly, with guilt cognitions related to hindsight bias, lack of justification, and perceived wrongdoing. We have cited this work [13,14] and work that has built on this model [37,56]. However, as with our comments on hindsight bias and Catch 22 dilemmas, the guilt components in this model can arise from survivor- and/or action-events (i.e., events underlying survivor- and action-guilt, as measured on CAPS). Thus, we have cited it as relevant work but not a work that leads to predictions regarding survivor- and action-guilt as predictors of PTSD symptom severity nor symptom cluster severity. Additionally, we also do not see where Kubany’s work leads to predictions regarding depression as a mediator of the relationship between guilt and PTSD symptom severity.

Aspects of Kubany’s work seems to us, at least in part, to be about promoting the expansion of the consideration of guilt in PTSD to go beyond survivor-guilt. We have also briefly discussed this history (paragraph beginning on Line 66) and the addition of action-guilt among the acknowledged “associated features” in PTSD. Within this context, we also have argued that testing for unique contributions of action- and survivor-guilt in predicting PTSD severity was a motivating factor for conducting this research, providing additional grounding of our motivation.

*8. The discussion of DMN as a potential mechanism is intriguing but speculative. Consider toning down or explicitly labeling it as a hypothesis for future research.*

We have explicitly labeled it as a hypothesis in need of future research (e.g., Line 328 “A mechanism by which guilt **might be** associated with PTSD…; Lines 336-339 “Although DMN dysfunctions **seems to be a candidate mechanism**, **direct research is needed** to examine DMN as a mediator of the associations between action- and survivor-guilt and PTSD…).

*9. Ensure all statistical tests are fully reported (e.g., effect sizes for non-significant results in Table 3).*

We have double-checked the data reported in all of the tables, including, the supplementary materials. All effect-size are reported. As noted in our response above, we have reformatted the tables for clarity.

*10. Clarify why certain covariates (e.g., combat exposure severity) were not included in analyses, as this could influence guilt-PTSD associations.*

The purpose of our study was to test for potential separable influences of survivor- and action-guilt related to the index trauma on PTSD symptom severity, including, potential unique associations with PTSD symptom clusters, and to examine potential mediation of the association by depression.

Indeed, meta-analysis has shown a range of pre-, peri-, and post trauma factors to be associated with PTSD and PTSD severity (e.g., [60]), to include, age, sex, race, SES, marital status, smoking & tobacco consumption, rank, branch of service, service occupation, number of deployments, length of deployment, combat exposure, discharging a weapon, seeing wounded/dead, trauma severity, adverse life events, psychological problems. However, in the meta-analyses [10,11] examining associations between guilt and PTSD symptom severity, limited moderators were examined. Among those, age was not found to be a significant moderator, and sex was found to be a significant moderator by Shi et al. [11] but not by Kip et al. [10].

We acknowledged in the limitations section that our study is not meant to be an exhaustive evaluation of all factors influencing PTSD severity and that other “pre- and peri-trauma factors” and “copying styles/strategies” (Lines 408-413) and that “future research including broader demographic assessments, a wider range of index traumas, and more detailed assessments of guilt will allow for more comprehensive evaluations of guilt profiles in PTSD in future research” (Lines 420-422).

Again, our interest was in potential unique associations between survivor- and action-guilt with PTSD symptom severity, and we did not have predictions regarding effects of the pre-, peri-, and post trauma factors listed above as moderators of the associations that we reported. However, based on the reviewer’s comment and the potential broader interests, we examined associations between the various demographic variables reported and the measures of interest in the study, and we referenced these analyses in the Limitations (lines 413-420) and included the results and summaries in the Supplementary Materials. As mentioned in the revised manuscript, none of the demographic variables were correlated with the primary measures of interest in the study, and thus, no further analyses were warranted.

Regarding the identified potential covariate of “combat exposure severity,” indeed, increased combat exposure has been shown to be a risk factor in developing PTSD (e.g., [60]), and we have also shown an association between combat exposure (i.e., Full Combat Exposure Scale) and PTSD Total severity in a prior publication on this sample of participants [61] (note that in that publication, survivor- and action-guilt were not examined).

For the revision, we examined the Full Combat Exposure Scale (FCES) as a potential mediator of the key results that we report in the manuscript on survivor- and action-guilt as predictors of PTSD Total and Avoidance/Numbing in PTSD. The FCES administered was the 34-item version in which participants endorsed experiencing combat events on a 5-point scale (0=never, 1=one time, 2=2-4 times, 3=5-9 times, and 4=10+ times), and FCES total was calculated by summing responses across the items. As shown in Supplementary Table S10, FCES total score was significantly correlated with CAPS Total (*r*=0.290), CAPS B (*r*=0.424), and Survivor-Guilt (*r*=0.296); however, FCES total score was not significantly correlated with CAPS C (*r*=0.195), Action-Guilt (*r*=0.200), nor QIDS (*r*=0.056).

Given the pattern of relationships, we examined FCES total as a potential mediator of the relationship between CAPS Total and Survivor-Guilt, although we also included Action-Guilt in the final step of this model. As shown in Supplementary Table S11, in Hierarchical Step 2, Survivor-Guilt significantly added to the variance accounted for (Δ*R^2^*=0.067, Δ*F*[2,52]=4.092, *p*<0.05) and remained a significant predictor of CAPS Total (*t*[52]=2.023, p<0.05) after adding it to the model following entering FCES, and then in Step 3, Action-Guilt was also found to be a significant predictor (Δ*R^2^* =0.102, *ΔF*[3,51]=6.996, *p*<0.05; *t*[51]=2.645, p<0.05). Finally, although the correlation between FCES and CAP C was not significant (*r*=0.195), we also included the results from the examination of FCES as a potential mediator in the Supplementary Materials (Table S12), both Survivor- and Action-Guilt remained significant contributing predictors of CAPS C after controlling for FCES. Thus, for both PTSD severity and avoidance and numbing severity, survivor- and action-guilt appear to have independent contributions from general combat exposure to PTSD total severity and avoidance/numbing severity.

We added a summary of these results in the limitations section (Lines 413-420):

“Furthermore, pre- and peri-trauma factors (e.g., personality [57], culture/social support [11], prior trauma [35,58], and coping style/strategies [35]) have been shown to be associated with guilt in PTSD. Demographic factors for the present sample also were examined for potential mediation of the observed effects (see Table S10), but only combat exposure (i.e., FCES Total [59]) was found to be significantly correlated with PTSD symptom severity (i.e., CAPS Total) [see also 60; 61] and survivor-guilt, but not avoidance/numbing (i.e., CAPS C) nor action-guilt. However, formal mediation testing via hierarchical regression, revealed that survivor-guilt remained a significant predictor of PTSD symptom severity after controlling for combat exposure (see Table S11; for CAPS C analysis, see Table S12).” In the Supplementary sections for Tables S10, S11, and S12, we also have provided summaries of the results from the analyses.

It makes sense that increased exposure to combat increases the chance of being exposed to an event or events leading to guilt and PTSD, but presumably, increased exposure to combat could also affect vulnerability a threshold for guilt and PTSD. However, our assessment of survivor- and action-guilt were from CAPS and, therefore, were specific to the “index trauma” reported and used for the CAPS assessment. As we have stated in the limitations section (Line 420) “future research including broader demographic assessments, a wider range of index traumas, and **more detailed assessments of guilt will allow for more comprehensive evaluations of guilt profiles** in PTSD in future research.”

*11. The clinical implications are well-articulated but could be expanded. For example, how might clinicians differentiate action-guilt from survivor-guilt in practice? Are there specific CPT techniques that could be highlighted?*

In CPT, differentiation of action- and survivor-guilt comes though the analysis of associations between activating events related to index trauma, maladaptive beliefs/cognitions, and consequences/feelings.

(Line 374-382) Thus, although survivor- and action-guilt severity are explicitly addressed on CAPS Associated Feature items, during CPT, clearer expressions of guilt associated with index trauma events and outcomes comes through discussions and targeted questions about the index trauma, stuck-points, and homework (e.g., expressions in the impact statement and worksheets, like, the A-B-C Worksheets focusing on connecting [A] Activating Event, [B] Belief/Cognitions, and [C] Consequences/Feelings and the Challenging Beliefs Worksheet that explicitly asks for ratings of emotions regarding stuck points), all making clearer to the therapist and client the associations between events and patterns of thinking contributing to survivor- and action-guilt.
