# Supplementary material for: Action-guilt, survivor-guilt, and depression in combat-related PTSD
Source: PLoS One. 2026 Jul 2;21(7):e0351689. doi: 10.1371/journal.pone.0351689 (PMC13327125; doi:10.1371/journal.pone.0351689)
Supplement: Peer review history

# Peer review: Decision Letter 0

**DOI:** 10.1371/journal.pone.0351689.r001

**Authors:** Giuseppe Marano

16 Dec 2024

PONE-D-23-42973

Depression mediates the association between action and survivor guilt in combat-related PTSD

PLOS ONE

Dear Dr. Motes,

Thank you for submitting your manuscript to PLOS ONE. After careful consideration, we feel that it has merit but does not fully meet PLOS ONE’s publication criteria as it currently stands. Therefore, we invite you to submit a revised version of the manuscript that addresses the points raised during the review process.

==============================

The reviewers have raised important points that will significantly enhance the quality of your manuscript. Please address each of their comments in detail and revise the manuscript accordingly. Furthermore, please pay close attention to the language used throughout the paper, ensuring that it is clear, concise, and grammatically correct.

To enhance the relevance and impact of your work, please consider incorporating more recent studies into your literature review. This will help to establish the novelty of your research and highlight its contributions to the field.

Please consider delving deeper into the implications of your findings, addressing potential limitations, and proposing future research directions that could build upon your work.

==============================

Please submit your revised manuscript by Jan 30 2025 11:59PM. If you will need more time than this to complete your revisions, please reply to this message or contact the journal office at plosone@plos.org. When you're ready to submit your revision, log on to https://www.editorialmanager.com/pone/ and select the 'Submissions Needing Revision' folder to locate your manuscript file.

Please include the following items when submitting your revised manuscript:

- A rebuttal letter that responds to each point raised by the academic editor and reviewer(s). You should upload this letter as a separate file labeled 'Response to Reviewers'.
- A marked-up copy of your manuscript that highlights changes made to the original version. You should upload this as a separate file labeled 'Revised Manuscript with Track Changes'.
- An unmarked version of your revised paper without tracked changes. You should upload this as a separate file labeled 'Manuscript'.

-->If you would like to make changes to your financial disclosure, please include your updated statement in your cover letter. Guidelines for resubmitting your figure files are available below the reviewer comments at the end of this letter.

If applicable, we recommend that you deposit your laboratory protocols in protocols.io to enhance the reproducibility of your results. Protocols.io assigns your protocol its own identifier (DOI) so that it can be cited independently in the future. For instructions see: https://journals.plos.org/plosone/s/submission-guidelines#loc-laboratory-protocols. Additionally, PLOS ONE offers an option for publishing peer-reviewed Lab Protocol articles, which describe protocols hosted on protocols.io. Read more information on sharing protocols at https://plos.org/protocols?utm_medium=editorial-email&utm_source=authorletters&utm_campaign=protocols.

We look forward to receiving your revised manuscript.

Kind regards,

Giuseppe Marano

Academic Editor

PLOS ONE

Journal requirements:

When submitting your revision, we need you to address these additional requirements.

1.  Please ensure that your manuscript meets PLOS ONE's style requirements, including those for file naming. The PLOS ONE style templates can be found at

https://journals.plos.org/plosone/s/file?id=wjVg/PLOSOne_formatting_sample_main_body.pdf  and

https://journals.plos.org/plosone/s/file?id=ba62/PLOSOne_formatting_sample_title_authors_affiliations.pdf

2. We note that the grant information you provided in the ‘Funding Information’ and ‘Financial Disclosure’ sections do not match.

When you resubmit, please ensure that you provide the correct grant numbers for the awards you received for your study in the ‘Funding Information’ section.

3. Thank you for stating the following in the Acknowledgments Section of your manuscript:

“This work was supported by Department of Defense GrantsW81XWH-11-2-0132 and W81XWH-18-1-0464 and by Texas Health and Human Services Commission HHSC Contract 529-14-0084-00001. We would like to thank all of the participants and family members who contributed time and effort to completing the study. We would also like to thank the collaborators and staff members who were involved with conducting the original clinical trial (Kozel et al., 2018).”

We note that you have provided funding information that is not currently declared in your Funding Statement. However, funding information should not appear in the Acknowledgments section or other areas of your manuscript. We will only publish funding information present in the Funding Statement section of the online submission form.

Please remove any funding-related text from the manuscript and let us know how you would like to update your Funding Statement. Currently, your Funding Statement reads as follows:

“The author(s) received no specific funding for this work.”

Please include your amended statements within your cover letter; we will change the online submission form on your behalf.

4. When completing the data availability statement of the submission form, you indicated that you will make your data available on acceptance. We strongly recommend all authors decide on a data sharing plan before acceptance, as the process can be lengthy and hold up publication timelines. Please note that, though access restrictions are acceptable now, your entire data will need to be made freely accessible if your manuscript is accepted for publication. This policy applies to all data except where public deposition would breach compliance with the protocol approved by your research ethics board. If you are unable to adhere to our open data policy, please kindly revise your statement to explain your reasoning and we will seek the editor's input on an exemption. Please be assured that, once you have provided your new statement, the assessment of your exemption will not hold up the peer review process.

5. Please include captions for your Supporting Information files at the end of your manuscript, and update any in-text citations to match accordingly. Please see our Supporting Information guidelines for more information: http://journals.plos.org/plosone/s/supporting-information.

6. Please review your reference list to ensure that it is complete and correct. If you have cited papers that have been retracted, please include the rationale for doing so in the manuscript text, or remove these references and replace them with relevant current references. Any changes to the reference list should be mentioned in the rebuttal letter that accompanies your revised manuscript. If you need to cite a retracted article, indicate the article’s retracted status in the References list and also include a citation and full reference for the retraction notice.

[Note: HTML markup is below. Please do not edit.]

Reviewers' comments:

Reviewer's Responses to Questions

**Comments to the Author**

1. Is the manuscript technically sound, and do the data support the conclusions?

The manuscript must describe a technically sound piece of scientific research with data that supports the conclusions. Experiments must have been conducted rigorously, with appropriate controls, replication, and sample sizes. The conclusions must be drawn appropriately based on the data presented.

Reviewer #1: Yes

Reviewer #2: Yes

Reviewer #3: Yes

**********

2. Has the statistical analysis been performed appropriately and rigorously?

Reviewer #1: Yes

Reviewer #2: Yes

Reviewer #3: Yes

**********

3. Have the authors made all data underlying the findings in their manuscript fully available?

The PLOS Data policy requires authors to make all data underlying the findings described in their manuscript fully available without restriction, with rare exception (please refer to the Data Availability Statement in the manuscript PDF file). The data should be provided as part of the manuscript or its supporting information, or deposited to a public repository. For example, in addition to summary statistics, the data points behind means, medians and variance measures should be available. If there are restrictions on publicly sharing data—e.g. participant privacy or use of data from a third party—those must be specified.

Reviewer #1: Yes

Reviewer #2: No

Reviewer #3: Yes

**********

4. Is the manuscript presented in an intelligible fashion and written in standard English?

PLOS ONE does not copyedit accepted manuscripts, so the language in submitted articles must be clear, correct, and unambiguous. Any typographical or grammatical errors should be corrected at revision, so please note any specific errors here.

Reviewer #1: Yes

Reviewer #2: Yes

Reviewer #3: Yes

**********

5. Review Comments to the Author

Please use the space provided to explain your answers to the questions above. You may also include additional comments for the author, including concerns about dual publication, research ethics, or publication ethics. (Please upload your review as an attachment if it exceeds 20,000 characters)

Reviewer #1: 1. Introduction is well narrated but only focus on that guilt is a consequence of PTSD symptoms, rather than a cause of PTSD symptoms, but also some other literature supports the opposite. So better to touch both ideas in order to send message for future study.

2. Better to show which guilty is in particular association with PTSD according to your finding?

3. Line 215-217 you explained the action guilty independently predicted PTSD symptom severity, so that better to explain whether the survivor guilt independently predicted PTSD symptom severity or not?

4. In discussion the claims must supported by the data so that it sounds better. Finally, you’re not adequately address the limitations of the study, I mean I can say there is no limitation of the study, so that please address it?

Reviewer #2: I would like to express my gratitude for the opportunity to review this manuscript. The study offers intriguing and valuable insights into its subject matter, which I find commendable. However, I believe it would greatly enhance the manuscript if the authors included a thorough discussion of the study's limitations. Addressing potential weaknesses or constraints of the research would provide a more balanced perspective.

Additionally, it would be beneficial to incorporate specific recommendations for future research based on the findings presented. This would not only guide subsequent studies but also demonstrate the practical applications of the research. Furthermore, discussing the theoretical implications of the study’s results would enrich the readers' understanding of its significance within the broader academic discourse.

Thank you once again for the opportunity to provide feedback on this manuscript.

Reviewer #3: The article addresses a significant and timely issue in the field of psychology, particularly concerning the mental health of combat veterans. The exploration of the interplay between guilt, depression, and PTSD is highly relevant, given the increasing recognition of the psychological impact of combat experiences. The findings contribute to the understanding of PTSD's etiology and treatment, aligning with current trends that emphasize the need for targeted interventions addressing trauma-related guilt and its consequences.

The title accurately reflects the study's focus on the mediating role of depression in the relationship between guilt (both action and survivor guilt) and PTSD symptoms. The literature review is comprehensive, citing relevant studies that establish the connections between guilt, depression, and PTSD. However, while the references are generally up-to-date, some could benefit from more recent studies to enhance the literature's breadth and depth.

The methodology section is detailed and provides essential information about the participant selection, assessment tools, and statistical analyses employed. However, it could benefit from a more explicit description of the exclusion criteria and the rationale behind the sample size. Additionally, while the use of the Clinician Administered PTSD Scale (CAPS) and the Quick Inventory of Depressive Symptomatology (QIDS) is appropriate, a brief discussion of their psychometric properties would strengthen the justification for their use.

The study excluded participants with certain psychiatric comorbidities and those who were not proficient in English. This could limit the generalizability of the findings to a broader population of veterans, particularly those who may have additional mental health challenges or language barriers.

The study is based on a secondary analysis of data collected for a different purpose (a clinical trial). This raises concerns about the original data's suitability for the current research questions. The lack of control over the initial data collection process may introduce biases or confounding variables that were not accounted for.

The scales used in the study are appropriate for measuring PTSD symptoms and depression. The CAPS is a well-established tool for assessing PTSD, and the QIDS is a reliable measure of depressive symptoms. However, the article could enhance its rigor by discussing the specific aspects of these scales that align with the study's focus on guilt and its effects

The statistical analyses employed, including hierarchical regression, are appropriate for the study's aims. The authors effectively demonstrate the mediating role of depression in the relationship between guilt and PTSD symptoms. However, a more detailed explanation of the assumptions underlying the regression analyses and how they were tested would improve the transparency of the statistical approach.

The values presented in the tables align with the interpretations provided in the text. The article could benefit from a more explicit connection between specific statistical values and their implications for the study's.

Moreover, the study does not appear to control for other potential confounding variables that could influence the relationships being studied, such as trauma history, social support, or coping strategies. These factors could significantly impact the severity of PTSD symptoms and the experience of guilt and depression.

The authors discuss the results in relation to previous literature, highlighting the unique contributions of action and survivor guilt to PTSD symptom severity. They effectively contextualize their findings within the broader research landscape. However, the discussion could be strengthened by addressing potential alternative explanations for the observed relationships and considering the implications of their findings for clinical practice.

The study acknowledges several limitations, including the small sample size and the cross-sectional design, which limits causal inferences. The authors recommend future research to explore longitudinal relationships and the effectiveness of guilt-targeting interventions. However, they could further address the potential impact of comorbid conditions beyond depression and suggest specific strategies for future studies to enhance generalizability.

Although the study suggests that guilt-targeting treatments may be beneficial, it does not provide specific recommendations for clinical practice or future research directions. A more thorough discussion of how these findings can be translated into effective interventions would enhance the study's impact.

**********

6. PLOS authors have the option to publish the peer review history of their article (what does this mean?). If published, this will include your full peer review and any attached files.

If you choose “no”, your identity will remain anonymous but your review may still be made public.

**Do you want your identity to be public for this peer review?**  For information about this choice, including consent withdrawal, please see our Privacy Policy.

Reviewer #1: **Yes:** Tadese Tefri Adugna

Reviewer #2: No

Reviewer #3: **Yes:** Dr. Syeda Rubab Aftab

**********

[NOTE: If reviewer comments were submitted as an attachment file, they will be attached to this email and accessible via the submission site. Please log into your account, locate the manuscript record, and check for the action link "View Attachments". If this link does not appear, there are no attachment files.]

While revising your submission, please upload your figure files to the Preflight Analysis and Conversion Engine (PACE) digital diagnostic tool, https://pacev2.apexcovantage.com/. PACE helps ensure that figures meet PLOS requirements. To use PACE, you must first register as a user. Registration is free. Then, login and navigate to the UPLOAD tab, where you will find detailed instructions on how to use the tool. If you encounter any issues or have any questions when using PACE, please email PLOS at figures@plos.org. Please note that Supporting Information files do not need this step.

# Author comment: Author response to Decision Letter 1

**DOI:** 10.1371/journal.pone.0351689.r002

16 Apr 2025

Reviewer #1

1. Introduction is well narrated but only focus on that guilt is a consequence of PTSD symptoms, rather than a cause of PTSD symptoms, but also some other literature supports the opposite. So better to touch both ideas in order to send message for future study.

We have clarified in the revision the possibility raised here in the Introduction, on Lines 90-101, relying on the review by Pugh el al. [1], and in the Discussion, particularly, the Lines 315-329 and in the added Limitations sections, particularly, Lines 380-386.

2. Better to show which guilty is in particular association with PTSD according to your finding?

We have clarified the findings in the Abstract, Lines 34-40; in the Results with summary statements on Lines 206-208, 215-218, 226-227, 248-250, 254-255, and 270-283; and the Discussion on Lines 289-292, 300-306, 307-309, 313-314, 352-356, and in the Conclusions.

3. Line 215-217 you explained the action guilty independently predicted PTSD symptom severity, so that better to explain whether the survivor guilt independently predicted PTSD symptom severity or not?

As noted in Reply #2, we have clarified the associations in the revision. Particularly, on the issue raised here, when action- and survivor-guilt were entered into the regression model, without QIDS, (Table 2 Model 1), both were significant predictors of CAPS Total. Thus, as we have clarified on Lines 206-208:

“Although Action-Guilt was the stronger predictor, Survivor-Guilt remained a significant predictor after entering Action-Guilt into the model first (i.e., statistically controlling for Action-Guilt). Thus, Action- and Survivor-Guilt both uniquely contributed to predicting CAPS Total.”

However, Survivor-Guilt was not a significant predictor of QIDS independent of Action-Guilt, as summarized on Lines 215-218:

“Thus, although Survivor-Guilt predicted depression on QIDS, the association was not independent of the association with Action-Guilt; whereas, Action-Guilt uniquely contributed to predicting depression on QIDS.”

Thus, when examining for mediation by QIDS, Action-Guilt was the predictor of interest. Similarly, in the PTSD Cluster analyses, Action-Guilt was the predictor of interest. We also have clarified this in the Discussion as noted in Reply #2 above.

4. Finally, you’re not adequately address the limitations of the study, I mean I can say there is no limitation of the study, so that please address it?

As described above in Reply #2, we have added a limitations section, Lines 357-386.

Reviewer #2

1. I believe it would greatly enhance the manuscript if the authors included a thorough discussion of the study's limitations. Addressing potential weaknesses or constraints of the research would provide a more balanced perspective.

As noted in Reviewer 1 Reply #4 above, we have added discussion of the limitations on Lines 357-386.

2. Additionally, it would be beneficial to incorporate specific recommendations for future research based on the findings presented. This would not only guide subsequent studies but also demonstrate the practical applications of the research. Furthermore, discussing the theoretical implications of the study’s results would enrich the readers' understanding of its significance within the broader academic discourse.

We have added discussion on possible future research in the Limitations section noted above, explicitly in Lines 377-380, and more implicitly Lines 304-306, 326-329, DMN discussion 330-341, and in the Conclusion regarding applications to therapy Lines 395-402.

Reviewer #3

1. The title accurately reflects the study's focus on the mediating role of depression in the relationship between guilt (both action and survivor guilt) and PTSD symptoms. The literature review is comprehensive, citing relevant studies that establish the connections between guilt, depression, and PTSD. However, while the references are generally up-to-date, some could benefit from more recent studies to enhance the literature's breadth and depth.

We have added many more recent references in the revision, notably, two meta-analyses examining guilt and PTSD: [10] Kip et al (2022) doi: 10.1017/S0033291722001866, and [11] Shi et al (2021) doi: 10.1016/j.janxdis.2021.102443.

2. The methodology section is detailed and provides essential information about the participant selection, assessment tools, and statistical analyses employed. However, it could benefit from a more explicit description of the exclusion criteria and the rationale behind the sample size. Additionally, while the use of the Clinician Administered PTSD Scale (CAPS) and the Quick Inventory of Depressive Symptomatology (QIDS) is appropriate, a brief discussion of their psychometric properties would strengthen the justification for their use.

We have included summaries of the psychometric properties of the measures in the Assessments sub-section, Lines 167-189.

We also have included more information on the inclusion/exclusion criteria and sample size for the original treatment trial, Lines 151-166. Because the present manuscript is a secondary analysis of data collected for that treatment trial, we did not obtain a priori minimum sample size estimate for this secondary analysis. We used all of the data that were available. However, the cited meta-analysis [11] reported a low-moderate to moderate (0.37<r<0.49) confidence interval for the association between combat-related guilt and PTSD symptom severity. Using the lower end, with r=0.37, alpha =0.05, and power=0.80, the minimum sample-size needed would be N=11. We have added discussion on the study not initially being powered for this secondary analysis to the Limitations section, Lines 360-362, in the revision.

3. The study excluded participants with certain psychiatric comorbidities and those who were not proficient in English. This could limit the generalizability of the findings to a broader population of veterans, particularly those who may have additional mental health challenges or language barriers.

As noted above, we have included a Limitations sections in the revision, and that Limitations section includes references to these issues.

4. The study is based on a secondary analysis of data collected for a different purpose (a clinical trial). This raises concerns about the original data's suitability for the current research questions. The lack of control over the initial data collection process may introduce biases or confounding variables that were not accounted for.

As noted above, we have included a Limitations section discussing the issues raised here.

5. The scales used in the study are appropriate for measuring PTSD symptoms and depression. The CAPS is a well-established tool for assessing PTSD, and the QIDS is a reliable measure of depressive symptoms. However, the article could enhance its rigor by discussing the specific aspects of these scales that align with the study's focus on guilt and its effects.

We have elaborated on issues relevant to the reviewer’s point in the Introduction and Discussion. Particularly, in the Introduction, Lines 52-69, we discuss findings on different guilt constructs in relation to PTSD. Then in Lines 70-89, we have provided more in-depth discussion on survivor- and action-guilt in PTSD. Then, in the Discussion, Lines 315-329, in particular, we discuss the present findings in relation to other research on guilt, depression, and PTSD. Finally, in the Limitations section of the Discussion, we also note the limitations of our work to action- and survivor-guilt and that a more comprehensive view of guilt in PTSD would require examining other components of guilt (e.g., distress, guilt cognitions, global guilt, hindsight bias, wrongdoing, lack of justification, etc.).

6. The statistical analyses employed, including hierarchical regression, are appropriate for the study's aims. The authors effectively demonstrate the mediating role of depression in the relationship between guilt and PTSD symptoms. However, a more detailed explanation of the assumptions underlying the regression analyses and how they were tested would improve the transparency of the statistical approach.

We have noted, on Lines 198-200, that there was no concerning evidence of regression residuals deviating from normality (i.e., showing outliers having undue influence, heteroscedasticity, and curvilinearity). The following figures show residual plots for PTSD Total and Avoidance/Numbing from the full model regression analyses and show PTSD Total and QIDS residuals plotted as functions of predicted values for the variables of interest. (See full Reply Letter for figures mentioned.)

7. The values presented in the tables align with the interpretations provided in the text. The article could benefit from a more explicit connection between specific statistical values and their implications for the study's. Moreover, the study does not appear to control for other potential confounding variables that could influence the relationships being studied, such as trauma history, social support, or coping strategies. These factors could significantly impact the severity of PTSD symptoms and the experience of guilt and depression.

As noted above in our reply to Reviewer 1 #2, we have added statements in the results to make more explicit the connections between the statistical values and their implications, in the Results with summary statements on Lines 206-208, 215-218, 226-227, 248-250, 254-255, and 270-283. Additionally, in the Limitations section, we have added discussion on the potential influences of trauma history, social support, and coping strategies.

8. The authors discuss the results in relation to previous literature, highlighting the unique contributions of action and survivor guilt to PTSD symptom severity. They effectively contextualize their findings within the broader research landscape. However, the discussion could be strengthened by addressing potential alternative explanations for the observed relationships and considering the implications of their findings for clinical practice.

We have provided elaboration on alternative explanations in the Discussion, Lines 315-329, by discussing previous research on the guilt, depression, and PTSD. We elaborate on studies treating PTSD and depression as separate constructs [37], separate but correlated constructs [36,38]. Additionally, in the Limitations section, we note issues with deriving causal inference from the current study and other data from correlational designs.

9. The study acknowledges several limitations, including the small sample size and the cross-sectional design, which limits causal inferences. The authors recommend future research to explore longitudinal relationships and the effectiveness of guilt-targeting interventions. However, they could further address the potential impact of comorbid conditions beyond depression and suggest specific strategies for future studies to enhance generalizability.

We have added discussion on these points to the added Limitations section.

10. Although the study suggests that guilt-targeting treatments may be beneficial, it does not provide specific recommendations for clinical practice or future research directions. A more thorough discussion of how these findings can be translated into effective interventions would enhance the study's impact.

As noted in other answers above, we have provided more on recommendations for future research in various parts of the Discussion section. With respect to treatments and potential research on treatments, we felt that it was necessary to be cautious with arguments regarding intervention specifics, because we are not presenting treatment data. However, we have added suggestions in the revision on Lines 395-402, particularly, elaborating on our previously short statement on “leverage points.”

Attachment Submitted filename: Reply.docx

# Peer review: Decision Letter 1

**DOI:** 10.1371/journal.pone.0351689.r003

**Authors:** Giuseppe Marano, Giuseppe Marano

13 May 2025

<div>PONE-D-23-42973R1

Action-Guilt, Survivor-Guilt, and Depression in Combat-Related PTSD

PLOS ONE

Dear Dr. Motes,

Thank you for submitting your manuscript to PLOS ONE. After careful consideration, we feel that it has merit but does not fully meet PLOS ONE’s publication criteria as it currently stands. Therefore, we invite you to submit a revised version of the manuscript that addresses the points raised during the review process.

==============================

I encourage you to follow the reviewers’ suggestions closely, as they provide valuable guidance for improving the manuscript. In particular, it would be important to further clarify both the strengths and limitations of your study in a dedicated section, offering a balanced and critical discussion. Additionally, please elaborate more clearly on how your findings can impact clinical practice — specifying potential applications or implications would significantly enhance the relevance and utility of your work for clinicians.

==============================

Please submit your revised manuscript by Jun 27 2025 11:59PM. If you will need more time than this to complete your revisions, please reply to this message or contact the journal office at plosone@plos.org. When you're ready to submit your revision, log on to https://www.editorialmanager.com/pone/ and select the 'Submissions Needing Revision' folder to locate your manuscript file.

Please include the following items when submitting your revised manuscript:

- A rebuttal letter that responds to each point raised by the academic editor and reviewer(s). You should upload this letter as a separate file labeled 'Response to Reviewers'.
- A marked-up copy of your manuscript that highlights changes made to the original version. You should upload this as a separate file labeled 'Revised Manuscript with Track Changes'.
- An unmarked version of your revised paper without tracked changes. You should upload this as a separate file labeled 'Manuscript'.

-->If you would like to make changes to your financial disclosure, please include your updated statement in your cover letter. Guidelines for resubmitting your figure files are available below the reviewer comments at the end of this letter.

If applicable, we recommend that you deposit your laboratory protocols in protocols.io to enhance the reproducibility of your results. Protocols.io assigns your protocol its own identifier (DOI) so that it can be cited independently in the future. For instructions see: https://journals.plos.org/plosone/s/submission-guidelines#loc-laboratory-protocols. Additionally, PLOS ONE offers an option for publishing peer-reviewed Lab Protocol articles, which describe protocols hosted on protocols.io. Read more information on sharing protocols at https://plos.org/protocols?utm_medium=editorial-email&utm_source=authorletters&utm_campaign=protocols.

We look forward to receiving your revised manuscript.

Kind regards,

Giuseppe Marano

Academic Editor

PLOS ONE

Journal Requirements:

Please review your reference list to ensure that it is complete and correct. If you have cited papers that have been retracted, please include the rationale for doing so in the manuscript text, or remove these references and replace them with relevant current references. Any changes to the reference list should be mentioned in the rebuttal letter that accompanies your revised manuscript. If you need to cite a retracted article, indicate the article’s retracted status in the References list and also include a citation and full reference for the retraction notice.

[Note: HTML markup is below. Please do not edit.]

Reviewers' comments:

Reviewer's Responses to Questions

**Comments to the Author**

1. If the authors have adequately addressed your comments raised in a previous round of review and you feel that this manuscript is now acceptable for publication, you may indicate that here to bypass the “Comments to the Author” section, enter your conflict of interest statement in the “Confidential to Editor” section, and submit your "Accept" recommendation.

Reviewer #2: (No Response)

Reviewer #3: All comments have been addressed

**********

2. Is the manuscript technically sound, and do the data support the conclusions?

The manuscript must describe a technically sound piece of scientific research with data that supports the conclusions. Experiments must have been conducted rigorously, with appropriate controls, replication, and sample sizes. The conclusions must be drawn appropriately based on the data presented.

Reviewer #2: Yes

Reviewer #3: Yes

**********

3. Has the statistical analysis been performed appropriately and rigorously?

Reviewer #2: Yes

Reviewer #3: Yes

**********

4. Have the authors made all data underlying the findings in their manuscript fully available?

The PLOS Data policy requires authors to make all data underlying the findings described in their manuscript fully available without restriction, with rare exception (please refer to the Data Availability Statement in the manuscript PDF file). The data should be provided as part of the manuscript or its supporting information, or deposited to a public repository. For example, in addition to summary statistics, the data points behind means, medians and variance measures should be available. If there are restrictions on publicly sharing data—e.g. participant privacy or use of data from a third party—those must be specified.

Reviewer #2: Yes

Reviewer #3: Yes

**********

5. Is the manuscript presented in an intelligible fashion and written in standard English?

PLOS ONE does not copyedit accepted manuscripts, so the language in submitted articles must be clear, correct, and unambiguous. Any typographical or grammatical errors should be corrected at revision, so please note any specific errors here.

Reviewer #2: Yes

Reviewer #3: Yes

**********

6. Review Comments to the Author

Please use the space provided to explain your answers to the questions above. You may also include additional comments for the author, including concerns about dual publication, research ethics, or publication ethics. (Please upload your review as an attachment if it exceeds 20,000 characters)

Reviewer #2: Thank you for the opportunity to review this manuscript. Although the authors have successfully addressed the majority of the previous concerns raised by the reviewers, there are still a few issues that require attention.

1. The authors should provide specific recommendations for each identified limitation.

2. It would be beneficial for the authors to include a distinct section outlining the practical and theoretical implications of the study, rather than incorporating them into the conclusion section.

Thank you.

Reviewer #3: All previous recommendations has been addressed. Few grammatical mistakes should be reviewed by the author.

**********

7. PLOS authors have the option to publish the peer review history of their article (what does this mean?). If published, this will include your full peer review and any attached files.

If you choose “no”, your identity will remain anonymous but your review may still be made public.

**Do you want your identity to be public for this peer review?**  For information about this choice, including consent withdrawal, please see our Privacy Policy.

Reviewer #2: No

Reviewer #3: No

**********

[NOTE: If reviewer comments were submitted as an attachment file, they will be attached to this email and accessible via the submission site. Please log into your account, locate the manuscript record, and check for the action link "View Attachments". If this link does not appear, there are no attachment files.]

While revising your submission, please upload your figure files to the Preflight Analysis and Conversion Engine (PACE) digital diagnostic tool, https://pacev2.apexcovantage.com/. PACE helps ensure that figures meet PLOS requirements. To use PACE, you must first register as a user. Registration is free. Then, login and navigate to the UPLOAD tab, where you will find detailed instructions on how to use the tool. If you encounter any issues or have any questions when using PACE, please email PLOS at figures@plos.org. Please note that Supporting Information files do not need this step.

# Author comment: Author response to Decision Letter 2

**DOI:** 10.1371/journal.pone.0351689.r004

30 Jul 2025

We would like to thank the reviewers for their time and attention in considering our manuscript. We are pleased that Reviewer 3 was satisfied with our responses and the previous revision and that Reviewer 2 was largely satisfied but added the following two suggestions.

1. The authors should provide specific recommendations for each identified limitation.

Regarding limitations, in the current revision, we have added the following statement on recommendations, “Thus, future research including broader demographic assessments, a wider range of index traumas, and more detailed assessments of guilt will allow for more comprehensive evaluations of guilt profiles in PTSD in future research.” Additionally, we have revised the paragraph to read, “Finally, given the correlational designs used in the present and previous studies, caution should be used in making strong causal inferences regarding guilt in PTSD. Guilt also has been proposed and modeled as a mediator between PTSD and suicidal ideation [58,59], for example. However, treatment studies also have shown reductions in guilt predict future reductions in PTSD severity [3,4,6,60,61], and meta-analysis has provided evidence of guilt predicting PTSD development longitudinally [10], with both findings providing additional evidence for directional inference in guilt contributing to PTSD severity.”

2. It would be beneficial for the authors to include a distinct section outlining the practical and theoretical implications of the study, rather than incorporating them into the conclusion section.

We added the following paragraph to the Discussion to further elaborate on the practical and theoretical implications (lines 353-381):

With interventions targeting trauma-related guilt having shown efficacy for reducing trauma-related guilt, depression, and PTSD symptom severity [2, 3, 5], the present findings suggest that targeting maladaptive cognitions separately underlying survivor- and action-guilt might have independent beneficial effects on PTSD symptom reduction. Although action- and survivor-guilt items on CAPS specifically address guilt frequency and intensity, cognitions and beliefs related to perceived culpability are components of guilt in PTSD (e.g., hindsight bias, preventability, violation of personal standards, and lack of justification2), and in CAPS-5 [53] guilt has been added to the diagnostic criteria (Item D4) on a symptom cluster assessing negative alterations in cognitions and mood that also includes items addressing cognitive distortions (Items D2 & D3). With both survivor- and action-guilt, the maladaptive cognitions and beliefs can be persecutorial and overaccommodative (e.g., with action-guilt, thinking that obsessive focus on wrongdoing will prevent future incidents or that living with never-ending guilt is simply the price for atonement or a way to honor those harmed; with survivor-guilt, obsessive focus on inequity in having survived when others did not and feeling less deserving of happiness [or living] due to the inequity and as a form of atonement) [54] but the unique contributions of survivor- and action-guilt to predicting PTSD symptom severity, observed in the present study, suggests some unique, mediating maladaptive cognitions can arise and contribute to PTSD symptom severity and can then be separately targeted for PTSD symptom reduction. In CPT integrating trauma accounts (CPT-A), the therapist and client use the index trauma account to gain an understanding the nature of a patient’s guilt and maladaptive cognitions, and the therapist can the target cognitive distortions and help the patient engage in cognitive restructuring and facilitate the creation of a narrative that fosters transition from avoidance/numbing to acceptance—key elements in CPT [54,55]. Furthermore, although both action- and survivor guilt were associated with avoidance/numbing in the present study, the observed unique association between action-guilt and avoidance/numbing and mediating effect of depression suggests that avoidance/numbing and comorbid depression might create greater therapeutic challenges with index-traumas triggering action-guilt, but the associations also suggest that successful therapeutic intervention changing maladaptive cognitions in action-guilt could more strongly affect avoidance/numbing which could then benefit other later-engaged therapeutic processes.

Attachment Submitted filename: response.docx

# Peer review: Decision Letter 2

**DOI:** 10.1371/journal.pone.0351689.r005

**Authors:** Giuseppe Marano, Giuseppe Marano, Giuseppe Marano

14 Aug 2025

PONE-D-23-42973R2

Action-Guilt, Survivor-Guilt, and Depression in Combat-Related PTSD

PLOS ONE

Dear Dr. Motes,

Thank you for submitting your manuscript to PLOS ONE. After careful consideration, we feel that it has merit but does not fully meet PLOS ONE’s publication criteria as it currently stands. Therefore, we invite you to submit a revised version of the manuscript that addresses the points raised during the review process.

It is recommended that all comments provided by the reviewers be carefully addressed. Particular attention should be given to the expansion of the discussion section, especially concerning the clinical implications of the findings. A more detailed consideration of how the results may be applied in clinical practice or inform future research is expected to strengthen the manuscript’s clarity and overall impact.

Please submit your revised manuscript by Sep 28 2025 11:59PM. If you will need more time than this to complete your revisions, please reply to this message or contact the journal office at plosone@plos.org. When you're ready to submit your revision, log on to https://www.editorialmanager.com/pone/ and select the 'Submissions Needing Revision' folder to locate your manuscript file.

Please include the following items when submitting your revised manuscript:

- A rebuttal letter that responds to each point raised by the academic editor and reviewer(s). You should upload this letter as a separate file labeled 'Response to Reviewers'.
- A marked-up copy of your manuscript that highlights changes made to the original version. You should upload this as a separate file labeled 'Revised Manuscript with Track Changes'.
- An unmarked version of your revised paper without tracked changes. You should upload this as a separate file labeled 'Manuscript'.

If you would like to make changes to your financial disclosure, please include your updated statement in your cover letter. Guidelines for resubmitting your figure files are available below the reviewer comments at the end of this letter.

-->If applicable, we recommend that you deposit your laboratory protocols in protocols.io to enhance the reproducibility of your results. Protocols.io assigns your protocol its own identifier (DOI) so that it can be cited independently in the future. For instructions see: https://journals.plos.org/plosone/s/submission-guidelines#loc-laboratory-protocols. Additionally, PLOS ONE offers an option for publishing peer-reviewed Lab Protocol articles, which describe protocols hosted on protocols.io. Read more information on sharing protocols at https://plos.org/protocols?utm_medium=editorial-email&utm_source=authorletters&utm_campaign=protocols.

We look forward to receiving your revised manuscript.

Kind regards,

Giuseppe Marano

Academic Editor

PLOS ONE

Journal Requirements:

1. If the reviewer comments include a recommendation to cite specific previously published works, please review and evaluate these publications to determine whether they are relevant and should be cited. There is no requirement to cite these works unless the editor has indicated otherwise.

2. Please review your reference list to ensure that it is complete and correct. If you have cited papers that have been retracted, please include the rationale for doing so in the manuscript text, or remove these references and replace them with relevant current references. Any changes to the reference list should be mentioned in the rebuttal letter that accompanies your revised manuscript. If you need to cite a retracted article, indicate the article’s retracted status in the References list and also include a citation and full reference for the retraction notice.

[Note: HTML markup is below. Please do not edit.]

Reviewers' comments:

Reviewer's Responses to Questions

**Comments to the Author**

1. If the authors have adequately addressed your comments raised in a previous round of review and you feel that this manuscript is now acceptable for publication, you may indicate that here to bypass the “Comments to the Author” section, enter your conflict of interest statement in the “Confidential to Editor” section, and submit your "Accept" recommendation.

Reviewer #2: All comments have been addressed

Reviewer #3: (No Response)

**********

2. Is the manuscript technically sound, and do the data support the conclusions?

The manuscript must describe a technically sound piece of scientific research with data that supports the conclusions. Experiments must have been conducted rigorously, with appropriate controls, replication, and sample sizes. The conclusions must be drawn appropriately based on the data presented.

Reviewer #2: Yes

Reviewer #3: Yes

**********

3. Has the statistical analysis been performed appropriately and rigorously?

Reviewer #2: Yes

Reviewer #3: Yes

**********

4. Have the authors made all data underlying the findings in their manuscript fully available?

The PLOS Data policy requires authors to make all data underlying the findings described in their manuscript fully available without restriction, with rare exception (please refer to the Data Availability Statement in the manuscript PDF file). The data should be provided as part of the manuscript or its supporting information, or deposited to a public repository. For example, in addition to summary statistics, the data points behind means, medians and variance measures should be available. If there are restrictions on publicly sharing data—e.g. participant privacy or use of data from a third party—those must be specified.

Reviewer #2: Yes

Reviewer #3: Yes

**********

5. Is the manuscript presented in an intelligible fashion and written in standard English?

PLOS ONE does not copyedit accepted manuscripts, so the language in submitted articles must be clear, correct, and unambiguous. Any typographical or grammatical errors should be corrected at revision, so please note any specific errors here.

Reviewer #2: Yes

Reviewer #3: Yes

**********

6. Review Comments to the Author

Please use the space provided to explain your answers to the questions above. You may also include additional comments for the author, including concerns about dual publication, research ethics, or publication ethics. (Please upload your review as an attachment if it exceeds 20,000 characters)

Reviewer #2: The authors have thoroughly and effectively addressed all concerns that were previously raised, demonstrating a commendable commitment to academic rigor. I am looking forward to the publication of this work, as it promises to contribute valuable insights to the field.

Thank you.

Reviewer #3: The study addresses a critical issue in PTSD research, focusing on guilt subtypes and their differential impacts, which has implications for targeted therapies. The use of hierarchical regression analyses is appropriate for testing mediation and unique contributions of guilt types. The inclusion of CAPS and QIDS measures is justified and aligns with standard practices in PTSD research. The findings are robust, showing independent contributions of action- and survivor-guilt to PTSD severity and mediation by depression. The nuanced analysis of symptom clusters (e.g., avoidance/numbing) adds depth. The manuscript thoroughly contextualizes the results within existing literature and proposes plausible mechanisms (e.g., DMN dysfunction). However, following point should be considering for revision

The manuscript is dense and could benefit from streamlining. For example, the introduction could be more concise, and the results section could better highlight key findings upfront.

Tables are detailed but could be simplified for readability (e.g., merging redundant models or adding a summary table).

The sample is predominantly male (93.4%) and from a specific combat era (OEF/OIF), which may limit generalizability. The authors should explicitly discuss this and caution against overgeneralizing to other populations (e.g., civilians, female veterans, or veterans from other conflicts).

The small sample size (N = 61) and secondary analysis design (with pre-existing data constraints) should be framed as limitations.

The study design is correlational, and while mediation is tested, causal claims should be tempered. The discussion appropriately notes this, but the abstract and introduction could more clearly state that causal inferences are speculative.

The introduction could better integrate the theoretical models of guilt in PTSD (e.g., Kubany’s cognitive model) to ground the hypotheses.

The discussion of DMN as a potential mechanism is intriguing but speculative. Consider toning down or explicitly labeling it as a hypothesis for future research.

Ensure all statistical tests are fully reported (e.g., effect sizes for non-significant results in Table 3).

Clarify why certain covariates (e.g., combat exposure severity) were not included in analyses, as this could influence guilt-PTSD associations.

The clinical implications are well-articulated but could be expanded. For example, how might clinicians differentiate action-guilt from survivor-guilt in practice? Are there specific CPT techniques that could be highlighted?

**********

7. PLOS authors have the option to publish the peer review history of their article (what does this mean?). If published, this will include your full peer review and any attached files.

If you choose “no”, your identity will remain anonymous but your review may still be made public.

**Do you want your identity to be public for this peer review?**  For information about this choice, including consent withdrawal, please see our Privacy Policy.

Reviewer #2: No

Reviewer #3: No

**********

[NOTE: If reviewer comments were submitted as an attachment file, they will be attached to this email and accessible via the submission site. Please log into your account, locate the manuscript record, and check for the action link "View Attachments". If this link does not appear, there are no attachment files.]

While revising your submission, please upload your figure files to the Preflight Analysis and Conversion Engine (PACE) digital diagnostic tool, https://pacev2.apexcovantage.com/. PACE helps ensure that figures meet PLOS requirements. To use PACE, you must first register as a user. Registration is free. Then, login and navigate to the UPLOAD tab, where you will find detailed instructions on how to use the tool. If you encounter any issues or have any questions when using PACE, please email PLOS at figures@plos.org. Please note that Supporting Information files do not need this step.

# Author comment: Author response to Decision Letter 3

**DOI:** 10.1371/journal.pone.0351689.r006

10 Dec 2025

We thank the editor and reviewers for their consideration of our manuscript for publication in PLOS One. We also appreciate the opportunity to address the latest comments, and we feel that the revisions made in response have strengthened the manuscript. Below are our responses to the comments. (Please note that the uploaded "reply" document includes formatting, figures, etc.).

1. The introduction could be more concise.

We have condensed the introduction, particularly, paragraph 2.

2. …the results section could better highlight key findings upfront

We have modified the results section to clarify the order of testing presented. For mediation testing, one must first show associations among the variables of interest [33]. The first paragraph in the results section reports on this testing, and we added the following line to clarify:

(Line 203) “Evaluations of associations between the variables of interest were conducted to obtain necessary evidence for mediation testing [33], that is assessments of unique associations between 1) CAPS Total and both Action- and Survivor Guilt, 2) CAPS Total and QIDS, and 3) QIDS and Action- and Survivor Guilt.”

We also added the following line as a summary to further clarify the importance of the results presented:

(Line 227) “Thus, the combinations of associations suggested possible mediation of the relationship between action-guilt and PTSD symptom severity by depression [33].”

3. Tables are detailed but could be simplified for readability (e.g., merging redundant models or adding a summary table).

We have revised the Tables to improve readability. In particular, we have reformatted the tables to highlight the steps in the regression hierarchical models. In the tables, we have separated analyses with solid lines and noted in the first column the criterion variable examined in the analysis. Then, we separated steps in each hierarchical model with dashed lines, numbered the results from the steps tested, and labeled the row showing the results from the “Final” model. Then, to highlight that the Beta-weights and t-values are from the “Final” model tested in the different analyses, we moved those statistics to the ends of the tables, shaded them in gray, and labeled the columns “Final Model Predictor Statistics” (see Table 2 example in the uploaded Reply and Tables in the revised manuscript).

We hope that these changes have also addressed the reviewer’s parenthetical comment about model redundancy. We are not reporting redundant models in Tables 2, 3, & 4. Arguably, there is redundancy in the regression model results reported in the Supplementary Materials because of a replication of patterns of statistics from prior steps in the hierarchical analyses, but the purpose of the Supplementary Tables is to report the complete sets of statistics from the hierarchical modeling steps, including, showing the changes to the regression weights with the addition of new variables to the model.

4. The sample is predominantly male (93.4%) and from a specific combat era (OEF/OIF), which may limit generalizability. The authors should explicitly discuss this and caution against overgeneralizing to other populations (e.g., civilians, female veterans, or veterans from other conflicts).

We stated this in the limitations section:

(Line 398) “The unique study selection pressures also might limit the generalization of the observed findings regarding guilt, depression, and PTSD. The data were from a sample of participants enrolled in a treatment trial for combat-related PTSD [7], that is, predominantly white male combat veterans from the OEF/OIF era of combat, with other specific demographic characteristics (e.g., English-speaking), who elected to enroll in the treatment trial.”

5. The small sample size (N = 61) and secondary analysis design (with pre-existing data constraints) should be framed as limitations.

We stated this in the limitations section:

(Line 395) “This was a secondary analysis of data collected for the treatment trial [6], and thus, for example, hypotheses were not formulated, measures used were not selected, and statistical power estimates were not obtained for this secondary analysis prior to data collection.”

6. The study design is correlational, and while mediation is tested, causal claims should be tempered. The discussion appropriately notes this, but the abstract and introduction could more clearly state that causal inferences are speculative.

As noted by the reviewer in this comment, in previous submissions, we explicitly stated this in the limitations section:

(Line 422) “Finally, given the correlational designs used in the present and previous studies, caution should be used in making strong causal inferences regarding guilt in PTSD.”

The only wording that we could find in our abstract suggesting “causal inference” is stated speculatively:

(Line 38) Thus, action- and survivor-guilt appear to have independent contributions to PTSD symptom severity, and action-guilt, more specifically, appears to affect avoidance and numbing severity in PTSD through depression.

The wording used in our introduction also is appropriately speculative:

(Line 85) Yet, although correlated, potential unique contributions of survivor- and action-guilt in predicting PTSD symptom severity have not been explored.

(Line 99) The present study aimed to further examine the potential role of guilt in PTSD by examining associations between trauma-related guilt, depression, and PTSD symptom severity.

We could not find any cases where we made “strong” causal claims, but if we have missed such cases, we are certainly willing to temper them as we have done with the statements referenced above and with our clear caveats regarding causal inferences that we have made in the limitations section (Lines 422-424).

7. The introduction could better integrate the theoretical models of guilt in PTSD (e.g., Kubany’s cognitive model) to ground the hypotheses.

It is not clear to us how this Kubany’s work would ground our hypotheses regarding survivor- and action-guilt, that is, beyond the referencing of that work that we have already put in the manuscript. We discuss the theoretical models of Pugh et al. [1] in the Introduction to ground the hypotheses (Lines 87-98), and this is in addition to our discussion of testing for potential unique associations of survivor- and action-guilt with PTSD severity (Lines 79-86). Additionally, we do reference Kubany’s work in the introduction [13,14] and discussion [65] sections, in addition to other related research on guilt profiles [21,22] and leverage points in therapy. However, based on our reading of Kubany’s work, the models do not lead to predictions regarding separable influences of survivor- and action-guilt, but maybe we are just not clear on the reviewer’s point here with respect to the relevance of Kubany’s work.

In our reading of Kubany’s work [65], “hindsight bias” is at the core of a form of “I should have known better” guilt; whereas, “Catch 22 dilemma” is at the core of a form of guilt arising from experiencing moral violations based actions in war referenced to “peacetime” moral values (i.e., war forces combatants to engage in behavior that would be considered “antithetically contrary to deeply ingrained family and societal values…[related to]…nonviolence and preservation of life”). However, as Kubany notes explicitly and in the anecdotes provided in [65], hindsight biases and Catch 22 dilemmas can arise from action/inaction events, survivor events, and their combinations. For example, in discussing hindsight bias, the three examples are on guilt arising from failures to act (i.e., action/inaction events) that led to deaths of compatriots (i.e., survivor events) (p 6), and under Catch 22, three of the anecdotes are on guilt arising from action events (i.e., killing combatants when not wanting to; killing children; using a deceased buddy as a shield) and the fourth in an action/inaction and survivor event (i.e., retreating under orders and surviving when a friend was killed) (p 8). In our reading, guilt coming from hindsight bias and Catch 22 dilemmas could be survivor- or action-guilt [65].

Kubany also proposed what we would argue is an elaboration on the above model with global guilt, distress, and guilt cognition components, particularly, with guilt cognitions related to hindsight bias, lack of justification, and perceived wrongdoing. We have cited this work [13,14] and work that has built on this model [37,56]. However, as with our comments on hindsight bias and Catch 22 dilemmas, the guilt components in this model can arise from survivor- and/or action-events (i.e., events underlying survivor- and action-guilt, as measured on CAPS). Thus, we have cited it as relevant work but not a work that leads to predictions regarding survivor- and action-guilt as predictors of PTSD symptom severity nor symptom cluster severity. Additionally, we also do not see where Kubany’s work leads to predictions regarding depression as a mediator of the relationship between guilt and PTSD symptom severity.

Aspects of Kubany’s work seems to us, at least in part, to be about promoting the expansion of the consideration of guilt in PTSD to go beyond survivor-guilt. We have also briefly discussed this history (paragraph beginning on Line 66) and the addition of action-guilt among the acknowledged “associated features” in PTSD. Within this context, we also have argued that testing for unique contributions of action- and survivor-guilt in predicting PTSD severity was a motivating factor for conducting this research, providing additional grounding of our motivation.

8. The discussion of DMN as a potential mechanism is intriguing but speculative. Consider toning down or explicitly labeling it as a hypothesis for future research.

We have explicitly labeled it as a hypothesis in need of future research (e.g., Line 328 “A mechanism by which guilt might be associated with PTSD…; Lines 336-339 “Although DMN dysfunctions seems to be a candidate mechanism, direct research is needed to examine DMN as a mediator of the associations between action- and survivor-guilt and PTSD…).

9. Ensure all statistical tests are fully reported (e.g., effect sizes for non-significant results in Table 3).

We have double-checked the data reported in all of the tables, including, the supplementary materials. All effect-size are reported. As noted in our response above, we have reformatted the tables for clarity.

10. Clarify why certain covariates (e.g., combat exposure severity) were not included in analyses, as this could influence guilt-PTSD associations.

The purpose of our study was to test for potential separable influences of survivor- and action-guilt related to the index trauma on PTSD symptom severity, including, potential unique associations with PTSD symptom clusters, and to examine potential mediation of the association by depression.

Indeed, meta-analysis has shown a range of pre-, peri-, and post trauma factors to be associated with PTSD and PTSD severity (e.g., [60]), to include, age, sex, race, SES, marital status, smoking & tobacco consumption, rank, branch of service, service occupation, number of deployments, length of deployment, combat exposure, discharging a weapon, seeing wounded/dead, trauma severity, adverse life events, psychological problems. However, in the meta-analyses [10,11] examining associations between guilt and PTSD symptom severity, limited moderators were examined. Among those, age was not found to be a significant moderator, and sex was found to be a significant moderator by Shi et al. [11] but not by Kip et al. [10].

We acknowledged in the limitations section that our study is not meant to be an exhaustive evaluation of all factors influencing PTSD severity and that other “pre- and peri-trauma factors” and “copying styles/strategies” (Lines 408-413) and that “future research including broader demographic assessments, a wider range of index traumas, and more detailed assessments of guilt will allow for more comprehensive evaluations of guilt profiles in PTSD in future research” (Lines 420-422).

Again, our interest was in potential unique associations between survivor- and action-guilt with PTSD symptom severity, and we did not have predictions regarding effects of the pre-, peri-, and post trauma factors listed above as moderators of the associations that we reported. However, based on the reviewer’s comment and the potential broader interests, we examined associations between the various demographic variables reported and the measures of interest in the study, and we referenced these analyses in the Limitations (lines 413-420) and included the results and summaries in the Supplementary Materials. As mentioned in the revised manuscript, none of the demographic variables were correlated with the primary measures of interest in the study, and thus, no further analyses were warranted.

Regarding the identified potential covariate of “combat exposure severity,” indeed, increased combat exposure has been shown to be a risk factor in developing PTSD (e.g., [60]), and we have also shown an association between combat exposure (i.e., Full Combat Exposure Scale) and PTSD Total severity in a prior publication on this sample of participants [61] (note that in that publication, survivor- and action-guilt were not examined).

For the revision, we examined the Full Combat Exposure Scale (FCES) as a potential mediator of the key results that we report in the manuscript on survivor- and action-guilt as predictors of PTSD Total and Avoidance/Numbing in PTSD. The FCES administered was the 34-item version in which participants endorsed experiencing combat events on a 5-point scale (0=never, 1=one time, 2=2-4 times, 3=5-9 times, and 4=10+ times), and FCES total was calculated by summing responses across the items. As shown in Supplementary Table S10, FCES total score was significantly correlated with CAPS Total (r=0.290), CAPS B (r=0.424), and Survivor-Guilt (r=0.296); however, FCES total score was not significantly correlated with CAPS C (r=0.195), Action-Guilt (r=0.200), nor QIDS (r=0.056).

Given the pattern of relationships, we examined FCES total as a potential mediator of the relationship between CAPS Total and Survivor-Guilt, although we also included Action-Guilt in the final step of this model. As shown in Supplementary Table S11, in Hierarchical Step 2, Survivor-Guilt significantly added to the variance accounted for (deltaR2=0.067, deltaF[2,52]=4.092, p<0.05) and remained a significant predictor of CAPS Total (t[52]=2.023, p<0.05) after adding it to the model following entering FCES, and then in Step 3, Action-Guilt was also found to be a significant predictor (deltaR2 =0.102, deltaF[3,51]=6.996, p<0.05; t[51]=2.645, p<0.05). Finally, although the correlation between FCES and CAP C was not significant (r=0.195), we also included the results from the examination of FCES as a potential mediator in the Supplementary Materials (Table S12), both Survivor- and Action-Guilt remained significant contributing predictors of CAPS C after controlling for FCES. Thus, for both PTSD severity and avoidance and numbing severity, survivor- and action-guilt appear to have independent contributions from general combat exposure to PTSD total severity and avoidance/numbing severity.

We added a summary of these results in the limitations section (Lines 413-420):

“Furthermore, pre- and peri-trauma factors (e.g., personality [57], culture/social support [11], prior trauma [35,58], and coping style/strategies [35]) have been shown to be associated with guilt in PTSD. Demographic factors for the present sample also were examined for potential mediation of the observed effects (see Table S10), but only combat exposure (i.e., FCES Total [59]) was found to be significantly correlated with PTSD symptom severity (i.e., CAPS Total) [see also 60; 61] and survivor-guilt, but not avoidance/numbing (i.e., CAPS C) nor action-guilt. However, formal mediation testing via hierarchical regression, revealed that survivor-guilt remained a significant predictor of PTSD symptom severity after controlling for combat exposure (see Table S11; for CAPS C analysis, see

Attachment Submitted filename: reply_r3.docx

# Peer review: Decision Letter 3

**DOI:** 10.1371/journal.pone.0351689.r007

**Authors:** Giuseppe Marano, Giuseppe Marano, Giuseppe Marano, Giuseppe Marano

1 Jun 2026

Action-Guilt, Survivor-Guilt, and Depression in Combat-Related PTSD

PONE-D-23-42973R3

Dear Dr. Motes,

We’re pleased to inform you that your manuscript has been judged scientifically suitable for publication and will be formally accepted for publication once it meets all outstanding technical requirements.

Within one week, you’ll receive an e-mail detailing the required amendments. When these have been addressed, you’ll receive a formal acceptance letter and your manuscript will be scheduled for publication.

An invoice will be generated when your article is formally accepted. Please note, if your institution has a publishing partnership with PLOS and your article meets the relevant criteria, all or part of your publication costs will be covered. Please make sure your user information is up-to-date by logging into Editorial Manager at Editorial Manager® and clicking the ‘Update My Information' link at the top of the page. For questions related to billing, please contact billing support.

If your institution or institutions have a press office, please notify them about your upcoming paper to help maximize its impact. If they’ll be preparing press materials, please inform our press team as soon as possible -- no later than 48 hours after receiving the formal acceptance. Your manuscript will remain under strict press embargo until 2 pm Eastern Time on the date of publication. For more information, please contact onepress@plos.org.

Kind regards,

Giuseppe Marano

Academic Editor

PLOS One

Additional Editor Comments (optional):

Reviewers' comments:

Reviewer's Responses to Questions

**Comments to the Author**

1. If the authors have adequately addressed your comments raised in a previous round of review and you feel that this manuscript is now acceptable for publication, you may indicate that here to bypass the “Comments to the Author” section, enter your conflict of interest statement in the “Confidential to Editor” section, and submit your "Accept" recommendation.

Reviewer #4: All comments have been addressed

**********

2. Is the manuscript technically sound, and do the data support the conclusions?

The manuscript must describe a technically sound piece of scientific research with data that supports the conclusions. Experiments must have been conducted rigorously, with appropriate controls, replication, and sample sizes. The conclusions must be drawn appropriately based on the data presented.

Reviewer #4: Yes

**********

3. Has the statistical analysis been performed appropriately and rigorously?

Reviewer #4: Yes

**********

4. Have the authors made all data underlying the findings in their manuscript fully available?

The PLOS Data policy requires authors to make all data underlying the findings described in their manuscript fully available without restriction, with rare exception (please refer to the Data Availability Statement in the manuscript PDF file). The data should be provided as part of the manuscript or its supporting information, or deposited to a public repository. For example, in addition to summary statistics, the data points behind means, medians and variance measures should be available. If there are restrictions on publicly sharing data—e.g. participant privacy or use of data from a third party—those must be specified.

Reviewer #4: Yes

**********

5. Is the manuscript presented in an intelligible fashion and written in standard English?

PLOS ONE does not copyedit accepted manuscripts, so the language in submitted articles must be clear, correct, and unambiguous. Any typographical or grammatical errors should be corrected at revision, so please note any specific errors here.

Reviewer #4: Yes

**********

6. Review Comments to the Author

Please use the space provided to explain your answers to the questions above. You may also include additional comments for the author, including concerns about dual publication, research ethics, or publication ethics. (Please upload your review as an attachment if it exceeds 20,000 characters)

Reviewer #4: Among a group of combat veterans, the current study demonstrated that survivor- and action-guilt were independent predictors of the severity of combat-related PTSD symptoms. Additionally, the study demonstrated that depression mediated the relationship between combat-related PTSD and action-guilt, and more precisely, depression mediated the relationship between action guilt and avoidance and numbing symptoms in PTSD. By demonstrating the distinction between the contributions of survivor- and action-guilt to PTSD severity, these findings advance our knowledge of trauma-related guilt in combat-related PTSD and advance the study of guilt profiles in PTSD. Potential leverage points in therapeutic interventions aimed at cognitive reappraisals can be found with the help of guilt assessments.

Well done. No furtehr changs are required.

**********

7. PLOS authors have the option to publish the peer review history of their article (what does this mean?). If published, this will include your full peer review and any attached files.

If you choose “no”, your identity will remain anonymous but your review may still be made public.

**Do you want your identity to be public for this peer review?**  For information about this choice, including consent withdrawal, please see our Privacy Policy.

Reviewer #4: **Yes:** Gyanesh Kumar Tiwari

**********

# Editor report: Acceptance letter

**DOI:** 10.1371/journal.pone.0351689.r008

**Authors:** Giuseppe Marano, Giuseppe Marano, Giuseppe Marano, Giuseppe Marano

PONE-D-23-42973R3

PLOS One

Dear Dr. Motes,

I'm pleased to inform you that your manuscript has been deemed suitable for publication in PLOS One. Congratulations! Your manuscript is now being handed over to our production team.

At this stage, our production department will prepare your paper for publication. This includes ensuring the following:

* All references, tables, and figures are properly cited

* All relevant supporting information is included in the manuscript submission,

* There are no issues that prevent the paper from being properly typeset

You will receive further instructions from the production team, including instructions on how to review your proof when it is ready. Please keep in mind that we are working through a large volume of accepted articles, so please give us a few days to review your paper and let you know the next and final steps.

Lastly, if your institution or institutions have a press office, please let them know about your upcoming paper now to help maximize its impact. If they'll be preparing press materials, please inform our press team within the next 48 hours. Your manuscript will remain under strict press embargo until 2 pm Eastern Time on the date of publication. For more information, please contact onepress@plos.org.

You will receive an invoice from PLOS for your publication fee after your manuscript has reached the completed accept phase. If you receive an email requesting payment before acceptance or for any other service, this may be a phishing scheme. Learn how to identify phishing emails and protect your accounts at https://explore.plos.org/phishing.

If we can help with anything else, please email us at customercare@plos.org.

Thank you for submitting your work to PLOS ONE and supporting open access.

Kind regards,

PLOS ONE Editorial Office Staff

on behalf of

Dr. Giuseppe Marano

Academic Editor

PLOS One
